# Characterization of the ‘Oat-Like Rice’ Caused by a Novel Allele *OsMADS1*^Olr^ Reveals Vital Importance of *OsMADS1* in Regulating Grain Shape in *Oryza sativa* L.

**DOI:** 10.1186/s12284-020-00428-x

**Published:** 2020-10-15

**Authors:** Penghui Li, Hui Li, Zhijian Liu, Yong Zhuang, Ming Wei, Yuanyang Gu, Yangxuan Liu, Xiuqiang Sun, Yuying Tang, Lu Yue, Longxiang Lu, Dagang Luo, Weizao Huang, Shengbin Tu, Songhu Wang

**Affiliations:** 1 Chengdu Institute of Biology, Chinese Academy of Sciences, Chengdu, 610041 China; 2 University of Chinese Academy of Sciences, Beijing, 100049 China; 3 Southeast Asia Biodiversity Research Institute, Chinese Academy of Sciences, Yezin, Nay Pyi Taw, 05282 Myanmar; 4 Crop Research Institute, Sichuan Academy of Agricultural Sciences, Chengdu, 610066 China

**Keywords:** Oat-like rice, *OsMADS1*^Olr^, Elongated leafy lemmas and paleae, Grain shape, Conjugated twin brown rice, Rice

## Abstract

**Background:**

Grain shape is a critical agronomic trait affecting grain yield and quality. Exploration and functional characterization of grain shape-related genes will facilitate rice breeding for higher quality and yield.

**Results:**

Here, we characterized a recessive mutant named Oat-like rice for its unique grain shape which highly resembles oat grains. The Oat-like rice displayed abnormal floral organs, an open hull formed by remarkably elongated leafy lemmas and paleae, occasionally formed conjugated twin brown rice, an aberrant grain shape and a low seed setting rate**.** By map-based cloning, we discovered that Oat-like rice harbors a novel allele of *OsMADS1* gene (*OsMADS1*^Olr^), which has a spontaneous point mutation that causes the substitution of an amino acid that is highly conserved in the MADS-box domain of the MADS-box family. Further linkage analysis indicated that the point mutation in the *OsMADS1*^Olr^ is associated with Oat-like rice phenotype, and expression analysis of the *OsMADS1* by qRT-PCR and GUS staining also indicated that it is highly expressed in flower organs as well as in the early stages of grain development. Furthermore, *OsMADS1*^Olr^-overexpressing plants showed similar phenotypes of Oat-like rice in grain shape, possibly due to the dominant negative effect. And *OsMADS1*-RNAi plants also displayed grain phenotypes like Oat-like rice. These results suggested that *OsMADS1*^Olr^ is responsible for the Oat-like rice phenotype including aberrant grain shape. Moreover, the expression levels of representative genes related to grain shape regulation were apparently altered in Oat-like rice, *OsMADS1*^Olr^-overexpressing and *OsMADS1*-RNAi transgenic plants. Finally, compared with Oat-like rice, *OsMADS1*^Olr^-overexpressing and *OsMADS1*-RNAi plants, mild phenotype of seed-specific *OsMADS1*-RNAi transgenic plants indicated that *OsMADS1* may has has a direct regulation role in grain development and the grain phenotypes of Oat-like rice, *OsMADS1*^Olr^-overexpressing and *OsMADS1*-RNAi plants are majorly caused by the abnormal lemma and palea development.

**Conclusions:**

Altogether, our results showed that grain shape and a low seed setting rate of the notable ‘Oat-like rice’ are caused by a spontaneous point mutation in the novel allele *OsMADS1*^Olr^. Furthermore, our findings suggested that *OsMADS1* mediates grain shape possibly by affecting the expression of representative genes related to grain shape regulation. Thus, this study not only revealed that *OsMADS1* plays a vital role in regulating grain shape of rice but also highlighted the importance and value of *OsMADS1* to improve the quality and yield of rice by molecular breeding.

## Background

Rice (*Oryza sativa* L.) is the main staple crops worldwide, feeding over half of the global population. The phenotypic traits of grain are the principal determinants of both rice yield and quality, which are the main targets of breeders (Wang et al. 2012). Grain shape, including grain length, width, and their ratio, not only is regarded as a significant indicator of appearance quality but also influences grain weight and nutritional quality (Lin and Wu 2003; Luo et al. 2004; Xu et al. 2004). In general, grain shape is one of most important evaluation criteria when breeders are developing rice varieties.

In rice, grain shape is closely related to spikelet development because the final grain shape is coordinately controlled by cell proliferation and cell expansion in the spikelet hull, which consists of a lemma and a palea (Li et al. 2018). The MADS-box genes are widely distributed transcription factors in plants and have a wide range of functions in controlling spikelet development. MADS-box proteins are characterized by the presence of a DNA binding domain known as the MADS-box domain, located in the N-terminal region of the protein. Additionally, the plant-specific MIKC-type MADS-box proteins, including OsMADS1, contain three additional domains followed by the highly conserved MADS domain, viz. a less-conserved **I**ntervening region (I region), a moderately conserved **K**eratin-like domain (K-box domain) mainly involved in heterodimerization, and a highly variable C-terminal region implicated in transcriptional activation and higher-order complex formation (Arora et al. 2007; Kumpeangkeaw et al. 2019).

Previous research has shown that *OsMADS1*, as a *SEPALLATA*-like MADS-box gene belongs to Class E gene in the ABCDE model of flower development (Hu et al. 2015). Additionally, several *OsMADS1* mutants such as *lhs1* (*leafy hull sterile1*) (Kinoshita et al. 1976; Jeon et al. 2000), *nsr* (*naked seed rice*) (Chen and Zhang 1980; Chen et al. 2006), *NF1019*, *ND2920*, *NE3043* and *NG778* (Agrawal et al. 2005), *osmads1-z* (Gao et al. 2010; Hu et al. 2015), *ohms1* (*open hull and male sterile 1*) (Sun et al. 2015) and *cyc15* (Zhang et al. 2018) display various degrees of defects in spikelets including of an open hull, overdeveloped lemma and palea, and homeotic conversion of some floral organs such as lodicules, stamens and carpels into lemma- and palea-like structures or organs. Furthermore, some genetic and molecular evidence has revealed that *OsMADS1* plays critical roles in floral organ development in rice by regulating the floral meristem specification and floral organ identity, particularly in the specification of the lemma and palea and acts as an inhibitor of overdevelopment of the lemma and palea (Jeon et al. 2000; Prasad et al. 2001; Chen et al. 2006; Wang et al. 2017).

On the one hand, it was reported that ectopic expression of *OsMADS1* in rice also results in multiple phenotypic variations in the panicles and grains of transgenic plants, including distorted panicles, decreased numbers of branches and spikelets per panicle, and elongated sterile lemmas in grains (Wang et al. 2017). These findings imply that *OsMADS1* not only regulates spikelet floral organs development, but it might also be related to panicle and grain development in rice. Furthermore, Khanday et al. (2013) showed that expression of *DEP1* (*DENSE AND ERECT PANICLE 1*), a gene encoding Gγ subunit protein, controls panicle morphology, grain numbers per panicle, grain size, grain weight and grain yield (Kong et al. 2007; Yan et al. 2007; Huang et al. 2009; Zhou et al. 2009; Sun et al. 2014; Li et al. 2019), with young developing panicles of *OsMADS1*-RNAi plants showing an 11.72 fold downregulation compared with wild type by Rice Affymetrix Gene Chips. Subsequently, Hu et al. (2015) also reported that expression of *DEP1* was significantly changed at spikelet stage 7–8 (Sp7–Sp8) of *osmads1-z* by whole-genome transcriptome analysis. In addition, Khanday et al. (2016) further reported that *DEP1* might be a target gene of OsMADS1 transcription factor by ChIP-seq (chromatin immunoprecipitation sequencing) and ChIP-qPCR analysis, although corresponding genetic evidence is lacking. These three studies provide clues that *OsMADS1* might be involved in the regulation of panicle and grain development by coordinating with other panicle and grain development-regulating genes such as *DEP1*.

In 2018 and 2019, four independent research groups uncovered the important roles of *OsMADS1* in regulating grain shape through interactions with G-protein subunits-encoding genes including *DEP1* and *GS3* (*Grain size 3*, encoding Gγ subunit protein) (Liu et al. 2018; Sun et al. 2018; Yu et al. 2018). *OsLG3b*, a QTL for long grains and higher yield, was reported to arise after domestication of tropical *japonica* and encode an alternatively spliced protein OsLG3b^SLG^ (Yu et al. 2018). Wang et al. (2019) dissected a heterotic QTL *GW3p6* which positively regulates grain length, 1000-grain weight and yield from the female line Guangzhan 63-4S through GradedPool-Seq mapping, and subsequently found that the *OsMADS1*^*GW3p6*^ gene corresponding to the QTL *GW3p6* was identical to the previously reported *OsLG3b*^SLG^ (Yu et al. 2018) and *OsMADS1*^lgy3^ gene (Liu et al. 2018). Moreover, Liu et al. (2018) reported that DEP1 and GS3 directly interact with the conserved K-box domain of OsMADS1. They serve as cofactors to enhance OsMADS1 transcriptional activity, thereby regulating grain shape and grain yield. Simultaneously, Sun et al. (2018) reported that three Gγ proteins, DEP1, GGC2 and GS3, antagonistically regulate grain size. DEP1 and GGC2, individually or in combination, increase grain length when in complex with the Gβ subunit protein, RGB1. Conversely, GS3 reduces grain length by competitively interacting with RGB1. Molecular and genetic evidence has shown that *OsMADS1* acts as a key downstream effector of G-protein βγ subunit proteins DEP1, GGC2, GS3 and RGB1 to regulate grain development (Liu et al. 2018).

Although the detailed molecular mechanism by which *OsMADS1* regulates grain shape is still largely unknown, researchers have found that many other genes regulate grain morphology by controlling cell division, cell proliferation, the ubiquitination pathway and the brassinosteroid signaling pathway, among others. *GW2* (*Grain width and weight 2*), encoding a RING-type E3 ubiquitin ligase, negatively regulates grain width and grain weight by suppressing cell division through targeting its substrate (s) to proteasomes for regulated proteolysis (Song et al. 2007). *GW5* (*qSW5, Grain width and weight 5*) encodes a calmodulin binding protein, which is also a negative regulator of grain width and weight by interacting with and repressing the kinase activity of rice GSK2 (Glycogen synthase kinase 2) in the brassinosteroid signalling pathway, subsequently influencing cell proliferation in spikelet hulls (Duan et al. 2017; Liu et al. 2017). *GS5* (*Grain size 5*) encodes a putative serine carboxypeptidase and functions as a positive regulator of grain size by regulating grain width, such that higher expression of *GS5* is correlated with a larger grain size (Li et al. 2011; Xu et al. 2015). Additionally, *GW8* (*OsSPL16*, *Squamosa promoter-binding protein-like 16*) encodes a SBP-domain transcription factor that is a positive regulator of cell proliferation, thus resulting in positive consequences for grain width and yield in rice (Wang et al. 2012; Wang et al. 2015). In addition, both *OsBU1* (*Brassinosteroid upregulated 1*) and *OsBC1* (*Brassinosteroid upregulated 1-like 1 complex 1*) encode basic helix-loop-helix (bHLH) transcriptional activators, which are positive regulators of the BR response and grain size (Tanaka et al. 2009; Jang et al. 2017).

To reveal the molecular mechanism by which *OsMADS1* regulates grain shape in rice, the functions of not only weak or mild alleles such as *OsLG3b*^SLG^ or *OsMADS1*^lgy3^ but also severe alleles of *OsMADS1* in grain development should be characterized and analyzed in details. In this study, we characterized a recessive mutant named Oat-like rice with a dramatic change in grain shape. Through map-base cloning, we found that Oat-like rice had a base substitution in the MADS domain of the *OsMADS1* gene and harbored a novel allelic *OsMADS1*^Olr^. The function of *OsMADS1*^Olr^ was analyzed by comprehensive phenotypic, genetic and molecular studies. Our results showed that *OsMADS1*^Olr^ not only regulates spikelet development but also plays a vital role in mediating grain shape in rice.

## Results

### The ‘Oat-Like Rice’ Is Named after the Unique Phenotypes in Grain Shape

**Fig. 1:**
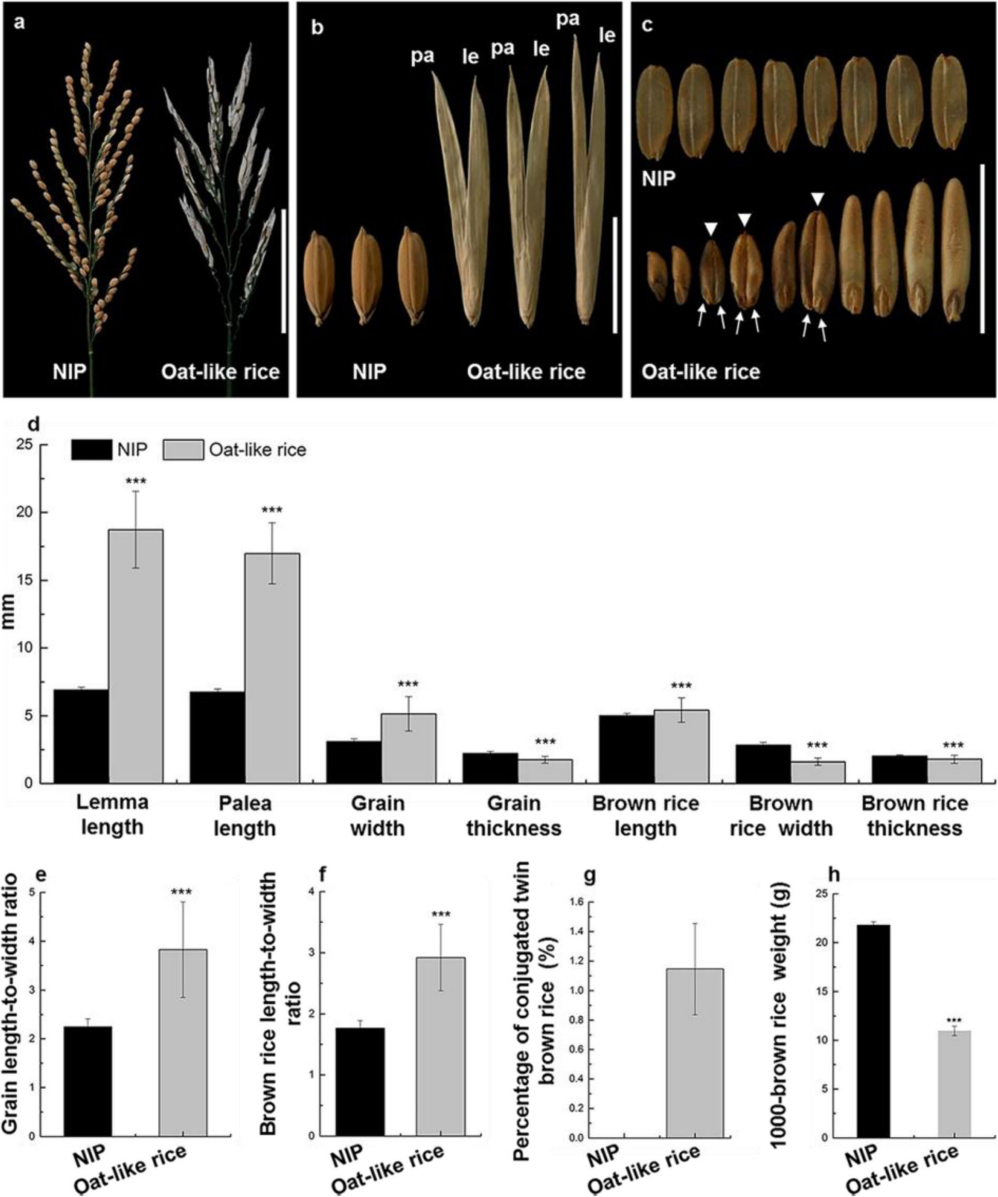
Typical morphological characteristics of Oat-like rice**. a** Panicle morphology of NIP (left) and Oat-like rice (right). le, lemma; pa, palea. **b** Comparison of grain shape between NIP (left) and Oat-like rice (right). **c** Morphological comparison of brown rice between NIP (up) and Oat-like rice (down). Arrowheads indicate conjugated twin brown rice; arrows indicate two embryos of a conjugated twin brown rice grain. **d** Comparison of lemma length, palea length, grain width, grain thickness, brown rice length, brown rice width and brown rice thickness between NIP and Oat-like rice. **e-h** Comparison of grain length-to-width ratio (**e**), brown rice length-to-width ratio (**f**), percentage of conjugated twin brown rice (**g**) and 1000-brown rice weight (**h**), between NIP and Oat-like rice. Bars: (**a**) 10 cm; (**b** and **c**) 1 cm. Data presented are mean values± SDs [*n* = 140 in **d**-**f**; *n* = 3 replicates (1000-brown rice for each replicate) in **g** and **h**.]. Student’s *t*-test: ^***^p < 0.001

The ‘Oat-like rice’ is a recessive mutant, which was originally discovered in the paddy field in 2001 for its recognizable phenotypes of off-white grains in panicles at the mature stage (Fig. 1a). Oat-like rice was characterized by a remarkably elongated leafy lemma and palea displaying a characteristic open hull (Fig. 1b), and occasional the formation of conjugated twin brown rice in the grains (1.15% ± 0.31%, Fig. 1c and g), which highly resembled oat grains (Fig. 1b and c). Therefore, it was known as ‘Oat-like rice’ for its unique phenotypes in grain shape by local farmers and rice breeders in the Sichuan province of China.

### Characterization of the Typical Morphological Characteristics of Oat-Like Rice

**Fig. 2:**
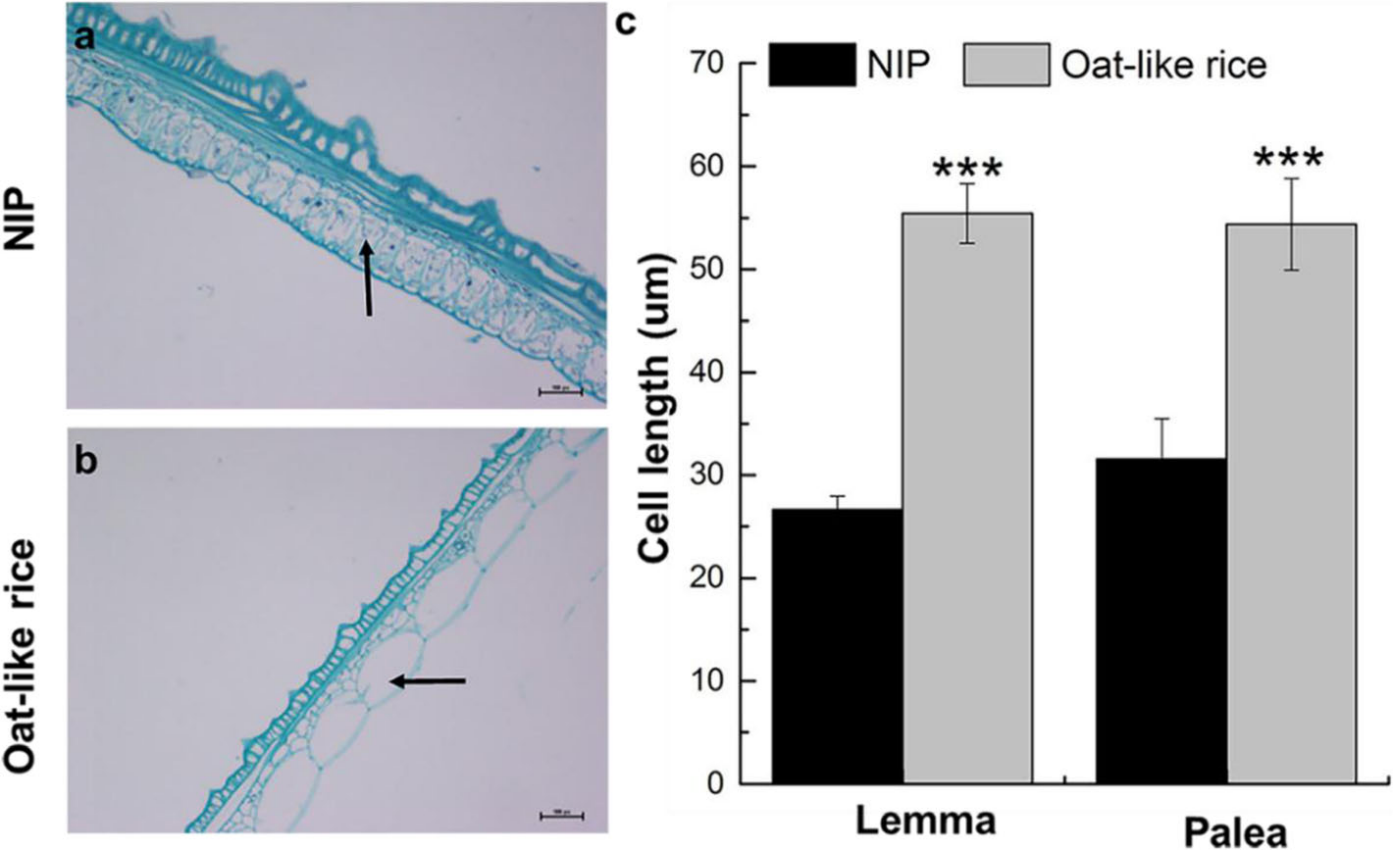
Histocytological analysis of spikelets hulls in NIP and Oat-like rice. **a** and **b** longitudinal sections at the middle of lemma in spikelets of NIP (**a**) and Oat-like rice (**b**). **c** Comparisons of cell length between NIP and Oat-like rice in the longitudinal sections at the middle of lemmas and paleae in spikelets. Arrows in **a** and **b** indicate parenchyma cells used for statistical analysis of cell length in (**c**). Bars: (**a** and **b**) 200 μm. Data presented are mean values± SDs [*n* = 27 replicates in c.]. Student’s t-test: ***p < 0.001

Although Oat-like rice exhibited some morphological differences from Nipponbare (NIP) including a distinct plant architecture, reduced plant height and total grain number per panicle, grain shape was the typical morphological characteristic used to distinguish Oat-like rice from NIP and other normal rice varieties (Additional file 1: Figures S1, S2 and Table S1, Fig. 1a-c, Additional file 2: Table S8). And the difference in plant architecture and plant height between Oat-like rice and NIP could be likely due to the difference of genetic backgrounds between the original wild type variety of Oat-like rice and NIP. As shown in Fig. 1c and g, some conjugated twin brown rice was occasionally observed in Oat-like rice, and Oat-like rice also had an extremely elongated lemma and palea compared with NIP. The length of the lemma and palea in Oat-like rice grains was 18.73 ± 2.83 mm and 16.98 ± 2.25 mm (Fig. 1d), respectively, which was approximately 1.7- and 1.5-fold longer than the lemma and palea, respectively, in NIP grains. To investigate the cause of the extremely elongated lemma and palea of Oat-like, we examined the lemma and palea of NIP and Oat-like grains by light microscopy. Longitudinal sections in the middle of lemma/palea showed that the parenchymal cells were much longer in Oat-like rice than in NIP (Fig. 2a and b). Further data analysis showed that the average cell length in the middle of the lemma and palea of Oat-like rice was 55.42 ± 1.28 mm and 54.37 ± 3.92 mm respectively, demonstrating an increased by approximately 108% and 72%, respectively, compared with NIP (Fig. 2c). Similar to the lemma and palea, the brown rice length of Oat-like rice was also significantly increased compared with that of NIP (Fig. 1d). Although the grains of Oat-like rice were extremely significantly wider than those of NIP due to the open hull formed by aberrant palea and lemma, the values of the grain thickness, brown rice width and brown rice thickness were extremely significantly lower than that of NIP (Fig. 1b and d). To summarize, Oat-like rice had a slender grain and brown rice, as indicated by the higher length-to-width ratio of the grain and brown rice (Fig. 1e and f). In addition, the 1000-brown rice weight of Oat-like rice was approximately half that of NIP (Fig. 1h), which maybe the result of difference in grain filling between them during the grain development after fertilization.

**Fig. 3:**
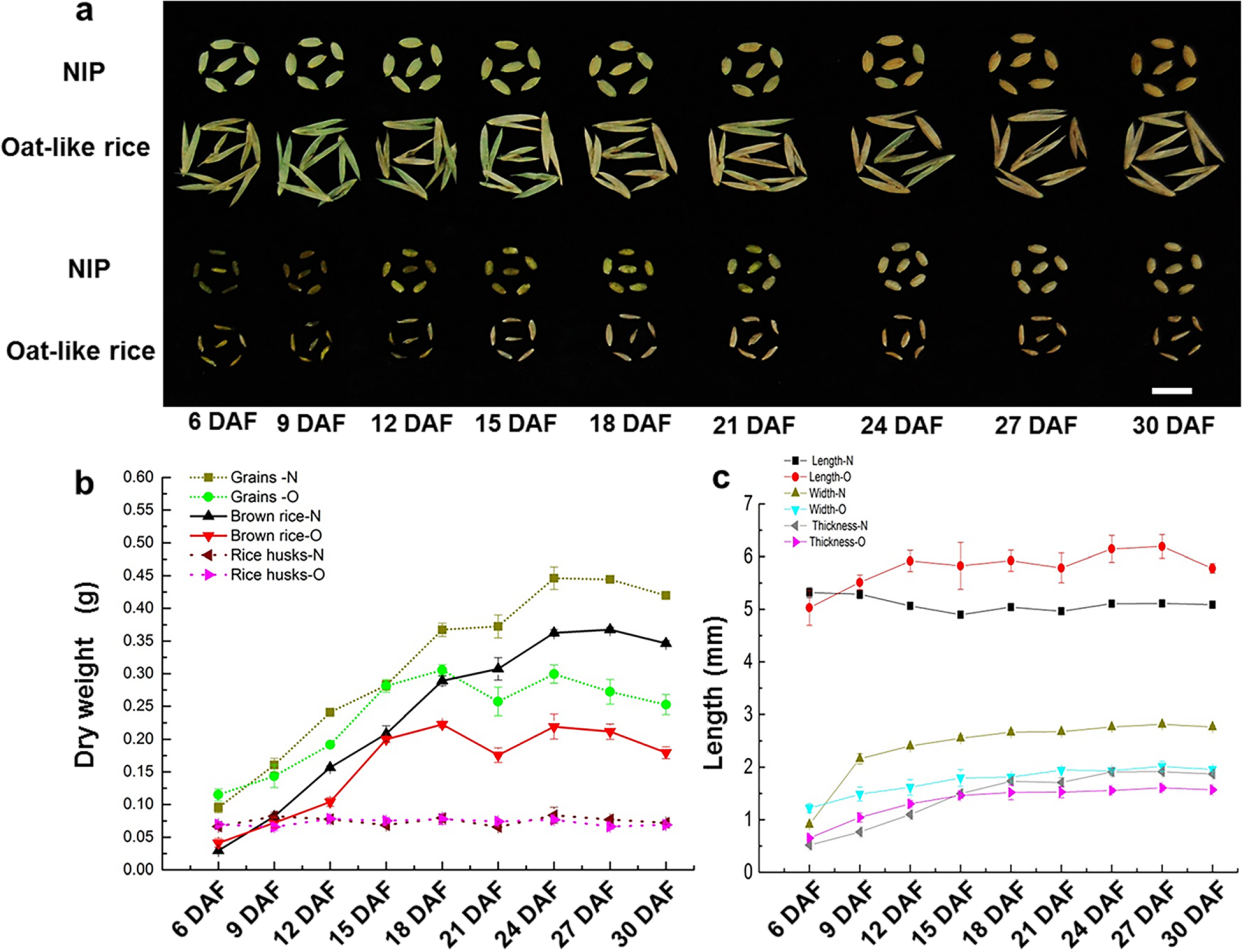
Time course analysis of the grain development between Oat-like rice and NIP. **a** Appearance and phenotypes of grains and brown rice of Oat-like rice and NIP at various developmental stages after fertilization. **b** Comparison of grain-filling process between Oat-like rice and NIP. **c** Comparison of brown rice length, brown rice width and brown rice thickness at various developmental stages after fertilization between Oat-like rice and NIP. N: NIP; O: Oat-like rice. Bar: (**a**) 5 mm. Data presented are mean values ± SDs [*n* = 3 replicates (each replicate consisting of 20 grains, brown rice and or pairs of lemma and palea) in **b**-**c**]

To further test this assumption, we performed time course analysis of grain development between Oat-like rice and NIP by measuring the values including dry weight, length, width and thickness of brown rice every 3 days from 6 to 30 DAF (Days After Fertilization). As shown in Fig. 3a and b, the trends in dry weight accumulation of both grains and brown rice between Oat-like rice and NIP were relatively similar in general. However, overall, Oat-like rice displayed an obviously slower grain-filling rate and lower dry weight accumulation than that of NIP, which eventually resulted in the significantly lower 1000-brown rice weight of Oat-like rice. In addition, during the grain filling process from 6 to 30 DAF, the dry weight of both grains and brown rice of Oat-like rice was markedly decreased at 21 DAF, which was contrary to that of NIP.

Moreover, changes in brown rice length, width and thickness between Oat-like rice and NIP were generally consist with their trends of dry weight accumulation, partly indicating by continuous increase of brown rice width and thickness from 6 to 27 DAF and subsequently slight decrease from 27 to 30 DAF (Fig. 3c). Besides, in Oat-like rice, the change curve of brown rice length was similar to that of dry weight accumulation, including a decrease at 21 DAF. In conclusion, the slower grain-filling rate and consequently lower 1000-brown rice weight of Oat-like rice suggested that its aberrant shape of brown rice may be also related to the affected grain filling during grain development. It should be noted that although some of the above phenotypic differences of grain shape and corresponding statistical significance tests between Oat-like rice and NIP may be affected by their difference of genetic backgrounds, the lemmas, paleae and grain length of Oat-like rice is extremely longer than other normal rice varieties which suggests that this phenotype should be caused by the spontaneous mutation in Oat-like rice rather than the difference of genetic backgrounds. We found that the plants showing Oat-like rice phenotype in F_2_ population only carried the homozygous mutation of *OsMADS1*^Olr^, while the normal plants carried the heterozygous *OsMADS1*^Olr^ or the homozygous *OsMADS1*. It suggested that the differences of grain phenotype between Oat-like rice and NIP might be not related to backgrounds. To further test this assumption, the grain length of 141 representative rice varieties or accessions including *Indica*, *Japonica*, *Aus*, *Aus/boro*, *Basmati/sadri*, and Intermediate type originated or collected from 57 countries is analyzed. As shown in Additional file 1: Figure S2 and Additional file 2: Table S8, the grain length of Oat-like rice is obvious longer than all these 141 rice varieties or accessions, which is consistent with the above assumption.

**Fig. 4:**
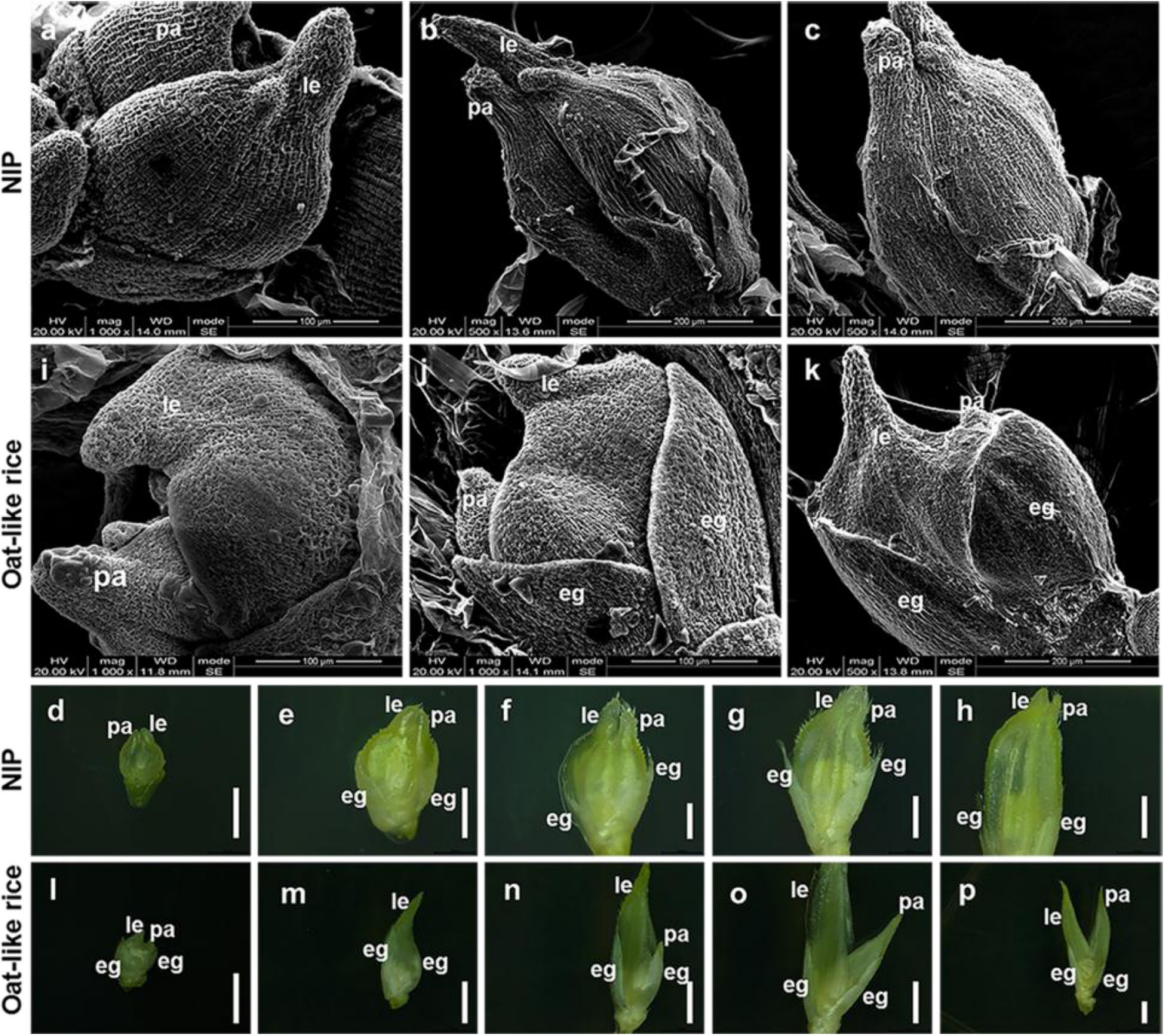
Observation and analysis of the representatively developmental stages of spikelets by SEM and dissecting microscope**. a** and **i** Spikelets of NIP (**a**) and Oat-like rice (**i**) at the stage of Sp8. **b-h** Spikelets of NIP; **j**-**p** Spikelets of Oat-like rice; **b-h**, **j-p** Spikelets after stage of Sp8. eg, empty glume; le, lemma; pa, palea; Bars: (**a, i** and **j**)100 μm, (**b, c** and **k**) 200 μm, (**d-h**, **l**-**p**) 1 mm

On the other hand, to investigate the cause of the open hull phenotype of Oat-like rice, we initially analyzed young spikelets at different developmental stages under a scanning electron microscope (SEM) and dissecting microscope. The lemma-palea showed an open structure at the end of Sp8 in both NIP and Oat-like rice (Fig. 4a and i) (Ikeda et al. 2004). In the subsequent developmental stages shortly after Sp8, the lemma and palea of NIP began to couple with each other and finally formed a closed spikelet (Fig. 4b-h). However, the lemma and palea of Oat-like rice remained open during all observed developmental stages of spikelets (Fig. 4j-p). As showed in Fig. 4j-p, the coordinate development of lemma and palea was disordered, as indicated by retarded palea relative to lemma and different dispositions and orientations between the lemma and palea, which caused the open hull phenotype of Oat-like rice.

In summary, Oat-like rice displayed a dramatic change in grain shape including occasionally formed conjugated twin brown rice and an open hull caused by uncoordinated development and elongation of lemmas and paleae.

### Aberrant Floral Organs and Defects in the Embryo Sac Affect the Seed Setting Rate of Oat-Like Rice

Apart from the open hull phenotype formed by extremely elongated lemma and palea, occasionally formed conjugated twin brown rice and a dramatic change in grain shape, an extremely low seed setting rate was another obvious abnormal characteristic of Oat-like rice. We initially analyzed the floral organ numbers, morphologies and structures in spikelets to investigate the cause of the low seed setting rate of Oat-like rice. As shown in Additional file 1: Figure S3a-c and j, the normal spikelet of NIP consisted of a pair of empty glumes, a lemma, a palea, a pair of lodicules, six stamens and a pistil. However, the spikelet of Oat-like rice displayed various variations in floral organ numbers, morphologies and structures, including altered floral organ numbers of empty glume, lemma, palea, lodicule, stamen, and pistil in a spikelet, as well as the number of stigma in a pistil (Additional file 1: Figure S3d-i, k-l and Table S2). Furthermore, some floral organs exhibited abnormal variations of morphologies, including leafy and extremely elongated lodicules, as well as conjugated pistils, which might cause the formation of twin brown rice in one grain (Additional file 1: Figure S3d-f, h, i and k). Nevertheless, there appeared to be no obvious difference in stamen morphologies between NIP and Oat-like rice (Additional file 1: Figure S3a, b and d-f). In addition, some floral organs of Oat-like rice exhibited aberrant structural variations, including hypertrophic lodicules and misshapen carpels (Additional file 1: Figure S3k and l). In summary, aberrant floral organs of Oat-like rice might not only impede normal pollination but also affect gamete fertility and finally the seed setting rate.

**Fig. 5:**
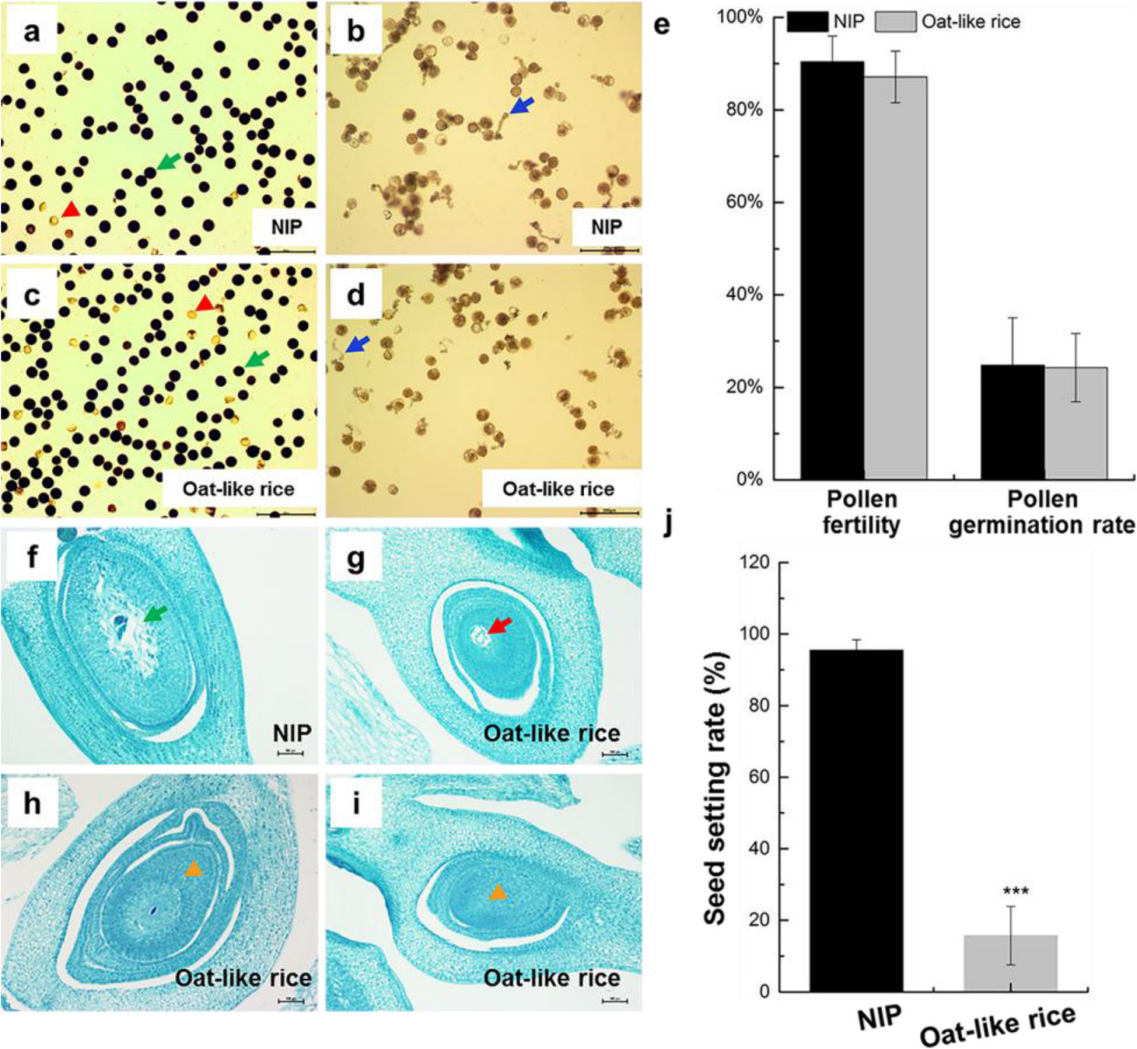
Fertility analysis of Oat-like rice. **a-d** Characterization of male fertility in NIP (**a** and **b**) and Oat-like rice (**c** and **d**). **a** and **c** I_2_-KI staining of pollen grains from NIP (**a**) and Oat-like rice (**c**). Red arrowheads indicate aborted pollen grains, green arrows indicate normal pollen grains (**a** and **c**). **b** and **d** Pollen in vitro germination assay of NIP (**b**) and Oat-like rice (**d**). Blue arrows indicate germinated pollen grains with pollen tubes (**b** and **d**). **e** Comparison of pollen fertility and pollen germination rate between NIP and Oat-like rice. **f-i** Comparison of mature embryo sacs between NIP (**f**) and Oat-like rice (**g**-**i**) by paraffin sections. **f-i** Longitudinal sections of mature carpels from NIP (**f**) and Oat-like rice (**g**-**i**). The green arrow in **f** indicates normal and mature embryo sac; the red arrow in **g** indicates shrinked embryo sac; orange arrowheads in **h** and **i** indicate that embryo sacs did not develop and were replaced with an abnormally proliferated nucellus. **j** Comparison of seed setting rate between NIP and Oat-like rice. Data presented are mean values ± SDs [*n* = 12 replicates in **e**; *n* = 24 replicates in **j**.]. Student’s *t*-test: ***p < 0.001. Bars: (**a**-**d**) 200 μm; (**f**-**i**) 100 μm

To further investigate this possibility, we analyzed the pollen vitality and germination ratio, morphologies and structures of embryo sac of Oat-like rice. There were no significant differences in the pollen vitality and pollen germination ratio between Oat-like rice and NIP (Fig. 5a-e), suggesting that the low seed setting rate of Oat-like rice might not be due to male gamete fertility. These results are consistent with the observation that stamen morphologies of Oat-like rice were apparently normal in comparison to NIP (Additional file 1: Figure S3a, b and d-f). However, about 6/7 abnormal carpels were observed in Oat-like rice, in which the embryo sac did not develop and was replaced with an abnormally proliferated nucellus (Fig. 5g-i). Thus, the low seed setting rate of Oat-like rice might be partly attributed to defects in the embryo sac of abnormal carpels (Fig. 5f-j). To summarize, the seed setting rate of Oat-like rice was related to aberrant floral organs and defects in the embryo sac.

### Map-Based Cloning of the *OsMADS1*^Olr^ Gene

**Fig. 6:**
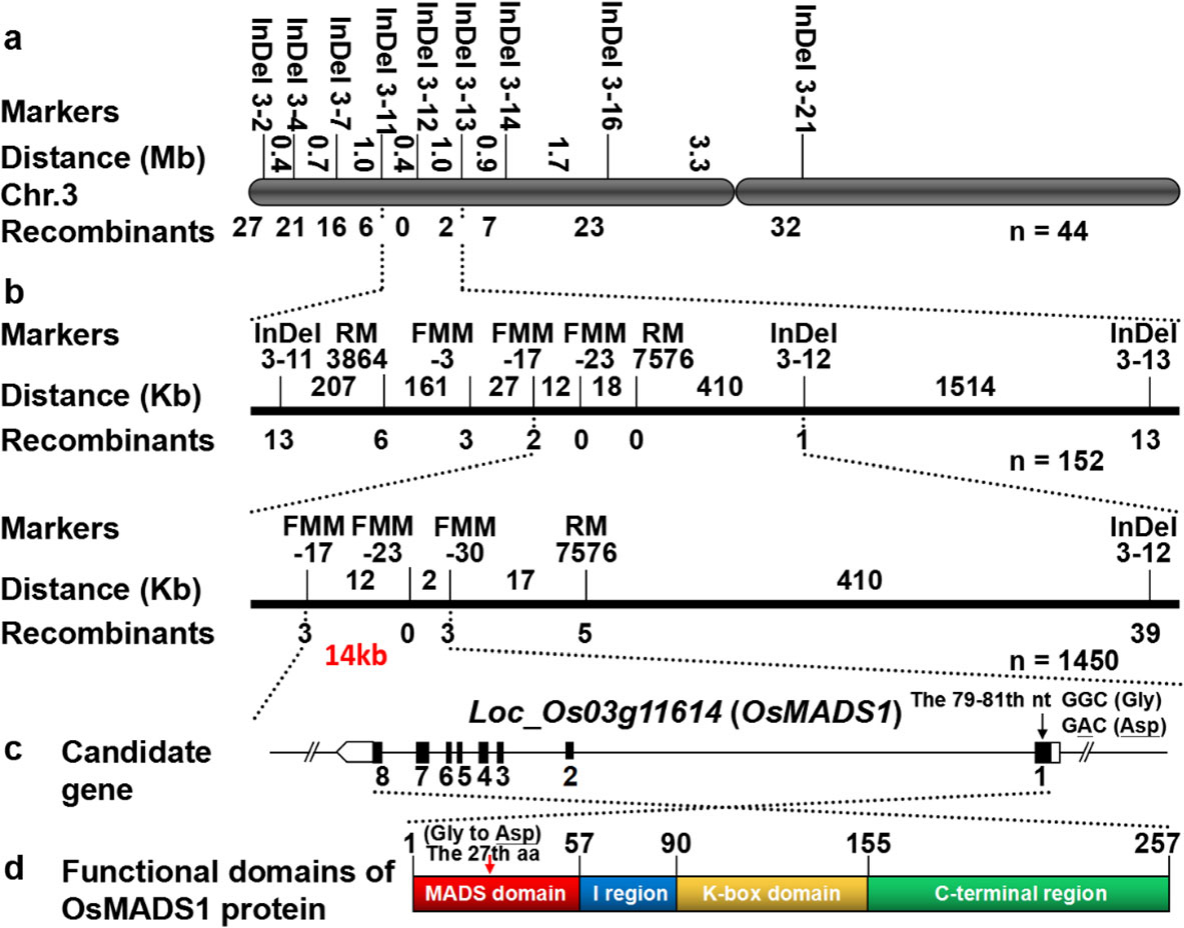
Map-Based Cloning of the *OsMADS1*^Olr^ gene. **a** Location of the *OsMADS1*^Olr^ gene on the long arm of chromosome 3. Numerals indicate physical distance between the adjacent markers. Numerals below the schematic diagram of chromosome 3 represent recombinants. **b** Fine mapping of the *OsMADS1*^Olr^ gene. Numerals above and below the bar represent physical distance and recombinants, respectively. **c** Schematic representation of gene structure of the *OsMADS1*. The nucleotide substitution site in *OsMADS1*^Olr^ is indicated by a black arrow. Exons are shown as closed boxes; introns are shown as straight lines; and untranslated regions (UTRs) are marked with open boxes. nt, nucleotide. **d** Schematic representation of OsMADS1 protein with functional domains. Functional domains of OsMADS1 with corresponding locations are shown. The amino acid substitution site in OsMADS1 protein is indicated by a red arrow. aa, amino acid

To clone the gene responsible for the unique grain shape in Oat-like rice, we constructed a mapping population derived from a cross between Oat-like rice and NIP. All the F_1_ plants showed a normal grain shape phenotype similar to NIP. The subsequent F_2_ population segregated into two phenotypes: the NIP phenotype and the Oat-like rice phenotype. However, the segregation ratio was approximately 1.50, which deviated from a typical ratio of 3:1 (χ^2^_3:1_ = 432.56 > χ^2^_0.05_ = 3.84, *p* < 0.001) (Additional file 1: Table S3), indicating that the phenotype of Oat-like rice might be controlled by a recessive quality trait locus with a segregation distortion. We initially found that the Oat-like rice locus was linked to the InDel (Insertion-Deletion) markers InDel 3–11 and InDel 3–13 on the long arm of chromosome 3 by bulked segregate analysis (BSA) (Fig. 6a). For preliminary mapping, 152 F_2_ individuals with the Oat-like phenotype were used, and new InDel markers were also developed based on the sequences of NIP and 93–11 (Additional file 1: Table S4). The Oat-like rice locus was first mapped to a 440-kb interval between markers FMM-17 and InDel 3–12 on chromosome 3 in rice (Fig. 6b). Then, we used 1450 F_2_ individuals with the Oat-like phenotype and developed additional new markers to finely map the locus and narrowed it down to an approximately 14-kb region (Fig. 6b). In the 14-kb interval, there was only one gene, *OsMADS1* (*LOC_Os03g11614*), predicted by the genome annotation database (http://www.gramene.org/), which contained eight exons and encoded a transcriptional regulator containing the MADS domain, I region, K-box domain and C-terminal region (Fig. 6c and d). A single nucleotide mutation of the 80th nucleotide (nt) in the first exon of *OsMADS1* was found through DNA sequencing (Fig. 6c). This point mutation from G to A caused a substitution from glycine (Gly) to aspartic acid (Asp) of the 27th amino acid in the MADS domain of the OsMADS1 protein in Oat-like rice (Fig. 6c and d). Some allelic mutations of *OsMADS1* have been reported to cause aberrant spikelets and floral organs in rice (Kinoshita et al. 1976; Chen and Zhang 1980; Jeon et al. 2000; Agrawal et al. 2005; Chen et al. 2006; Gao et al. 2010; Hu et al. 2015; Sun et al. 2015; Zhang et al. 2018)*.* We compared the mutation site in the *OsMADS1* gene between Oat-like rice and other reported mutants, and we found that the point mutation of Oat-like rice differed from the previously identified mutations in the *OsMADS1* gene. Therefore, the Oat-like rice harbors a novel allele of the *OsMADS1* gene, which was designated *OsMADS1*^Olr^.

### The Mutation Site in *OsMADS1*^Olr^ Is Associated with the Oat-Like Rice Phenotype and the Corresponding Wild-Type Amino Acid Is Highly Conserved in MADS-Box Proteins in Rice

To investigate if the mutation in *OsMADS1*^Olr^ is responsible for the Oat-like rice phenotype, we initially performed a sequence alignment of exon 1 of the *OsMADS1* gene among Oat-like rice and thirteen representatively normal rice varieties including *japonica* rice NIP and *Indica* rice 93–11. We found that only Oat-like rice carried the mutation in *OsMADS1*^Olr^, while all the other thirteen representative rice varieties with a normal grain shape carried the wild type nucleotide of *OsMADS1* (Additional file 1: Figure S4). Besides, We found that the plants showing Oat-like rice phenotype in F_2_ population only carried the homozygous mutation of *OsMADS1*^Olr^, while the normal plants carried the heterozygous *OsMADS1*^Olr^ or the homozygous *OsMADS1*. These results indicated that the mutation in *OsMADS1*^Olr^ was associated with the Oat-like rice phenotype.

Furthermore, the MADS-box domain of OsMADS1 protein was responsible and indispensable for DNA binding between OsMADS1 and its target genes (Arora et al. 2007). To explore the conservation of the substitution site of *OsMADS1*^Olr^ in the rice MADS-box family, we compared the protein sequence of the MADS-box domain among the 74 MADS-box family members in rice obtained from the Plant Transcription Factor Database (Plant TFDB). By sequence alignment, we found that the amino acid (Gly) and the corresponding nucleotide (corresponding G) in the substitution site was highly conserved in MADS-box family members in rice (Additional file 1: Figure S5 and Fig. 6c and d). Interestingly, this wild-type amino acid (Gly) in the MADS-box domain is not only highly conserved in MADS-box family members in rice, but is also highly conserved in the MADS-box transcription factors from plants, yeast, frog and human, including AP1 (APETALA1) and CAL (CAULIFLOWER) in *Arabidopsis*, and SRF (Serum Response Factor) in human. And the Gly was important for the DNA binding of MADS-box domain (Pellegrini et al. 1995). AP1 and CAL are two partially redundant MADS-box genes. Both genes are expressed in young flower primordia and involved in regulation of specifying, identity and development of the floral meristem in *Arabidopsis*. The *ap1–2* single mutant of *AP1* and *ap1-1 cal-2* double mutant of *AP1* and *CAL* exhibited phenotypic alterations in flowers including abnormally proliferous inflorescence, axillary flowers and aberrant floral organs due to a partial conversion of floral meristems into inflorescences. Coincidentally, mutations in AP1^ap1–2^ and CAL^cal-3^ proteins are exactly the same to those in mutated OsMADS1^Olr^ protein (Mandel et al. 1992; Kempin et al. 1995). Therefore, the wild-type glycine in the substitution site may be essential for the normal DNA binding functions of MADS-box family proteins and amino acid substitution in this site of OsMADS1 may cause the abnormal grain shape in Oat-like rice.

### Expression Analysis Suggests that *OsMADS1* Is Involved in Grain Development

**Fig. 7:**
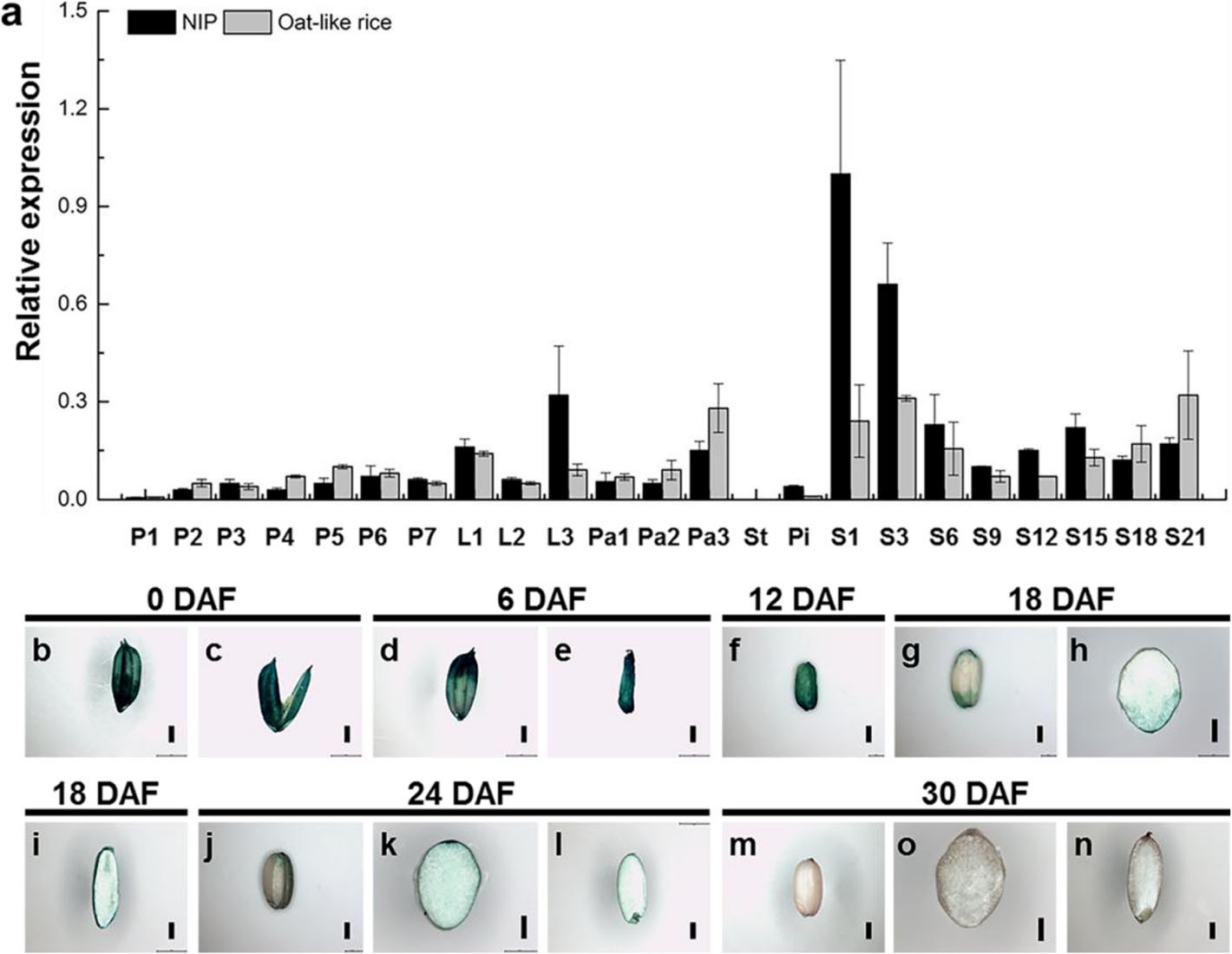
Tissue-specific RNA expression pattern of the *OsMADS1* and *OsMADS1*^Olr^. **a** Relative expression levels of the *OsMADS1* and *OsMADS1*^Olr^ in NIP and Oat-like rice, respectively. Samples from panicles, floral organs and grains at representative developmental stages were analyzed. P1, 0–3 mm panicles; P2, 3–15 mm panicles; P3, 15–50 mm panicles; P4, 50–100 mm panicles; P5, 100–150 mm panicles; P6, 150–200 mm panicles; P7, panicles longer than 200 mm; L1, lemmas of spikelets from 100 to 150 mm panicles; L2, lemmas of spikelets from 150 to 200 mm panicles; L3, lemmas of spikelets from panicles longer than 200 mm; Pa1, paleae of spikelets from 100 to 150 mm panicles; Pa2, paleae of spikelets from 150 to 200 mm panicles; Pa3, paleae of spikelets from panicles longer than 200 mm; St, stamens of spikelets before anthesis from panicles longer than 200 mm; Pi, pistils of spikelets before anthesis from panicles longer than 200 mm; S1, 1 DAF pistils and hulls; S3, 3 DAF grains; S6, 6 DAF grains; S9, 9 DAF grains; S12, 12 DAF grains; S15, 15 DAF grains; S18, 18 DAF grains; S21, 21 DAF grains. DAF, Days After Fertilization. The value of *OsActin* mRNA was used as an internal control for data normalization, and the expression level of *OsMADS1* in S1 (1 DAF pistils and hulls) of NIP were set as 1.0. Values are means ± SDs of three replicates. **b-n** Histochemical staining analysis of *GUS* expression driven by *OsMADS1* promoter in transgenic grains at representative developmental stages. **b** and **c** A mature spikelet (**b**) and hull of a mature spikelet (**c**). **d** A 6 DAF grain. **e-g, j** and **m** Brown rice. **h, k** and **o** The transverse section of brown rice. **i, l** and **n** Longitudinal section of brown rice. Bars: (**b**-**n**) 1 mm

Previous reports have indicated that *OsMADS1* is mainly expressed in the inflorescence, spikelets and during grain development (Chung et al. 1994; Arora et al. 2007; Liu et al. 2018; Zhang et al. 2018). To investigate whether *OsMADS1*^Olr^ is expressed and functional, and if *OsMADS1*^Olr^ has the similar expression pattern of *OsMADS1* in NIP, we analyzed the expression patterns of *OsMADS1* and *OsMADS1*^Olr^ in NIP and Oat-like rice respectively during floral and grain development. Our quantitative real time PCR (qRT-PCR) results showed that *OsMADS1* and *OsMADS1*^Olr^ had similar expression patterns, and the average expression of both *OsMADS1* and *OsMADS1*^Olr^ was higher in grains than in inflorescences and floral organs. Additionally, we detected the strongest expression in the early stages of grain development, especially in 1 DAF pistils and hulls and 3 DAF grains (Fig. 7a). Furthermore, expression of *OsMADS1* or *OsMADS1*^Olr^ was also abundant in elongating lemmas (L1), mature lemmas (L3) and paleae (Pa3) (Fig. 7a). *OsMADS1* or *OsMADS1*^Olr^ was expressed in pistils, but expression was not detected in stamens (Fig. 7a). Considering both spikelets and grains of Oat-like rice displayed abnormal morphologies, these results implies that *OsMADS1* not only plays a vital role in regulating spikelet development, but also may be functional in regulating grain development.

To further analyze the detailed tissue-specific expression of *OsMADS1*, we analyzed *GUS* expression driven by the native *OsMADS1* promoter in transgenic grains. The GUS staining showed that *OsMADS1* was highly expressed in grains at 0 DAF, 6 DAF and 12 DAF. Then, its expression was significantly reduced and was not detected in fully mature grains (Fig. 7b-n). Overall, *OsMADS1* was mainly expressed in the lemma, palea and grain epidermis during the early stage of grain development and in the embryo, endosperm and aleurone layer. Moreover, we subsequently checked and analyzed the tissue-specific expression pattern of *OsMADS1* based on the microarray data released in the ePlant Rice database (http://bar.utoronto.ca/eplant_rice/) and the Rice Expression Profile Database (RiceXPro) (https://ricexpro.dna.affrc.go.jp/category-select.php), respectively (Itoh et al. 2005; Waese et al. 2017). *OsMADS1* was also mainly expressed in inflorescences, spikelets, floral organs and grains including embryo, endosperm and aleurone layer, whereas expressions of *OsMADS1* were not detected or negligible in the vegetative tissues and organs such as roots, stems and leaves (Additional file 1: Figure S8a and c). Taken together, this result was consistent with our qRT-PCR and GUS staining results, and the expression of *OsMADS1* in grains by qRT-PCR and GUS staining analysis were generally comparable. Collectively, *OsMADS1* was highly expressed during the early stage of grain development and might play a role in the grain development.

### *OsMADS1*^Olr^ Is Responsible for the Oat-Like Rice Phenotype

Partly because of its genetic background of *incica* rice, the Oat-like rice calli is very hard for genetic transformation to introduce the wild-type *OsMADS1* into the Oat-like rice to verify whether *OsMADS1*^Olr^ was responsible for the Oat-like rice phenotype by complementation experiment. And previous study reported that overexpression of mutated version of the MADS-box gene in transgenetic plants caused dominant-negative effect, such as the mutated *AGAMOUS* (*AG*) gene lacking the C-terminal region in *Arabidopsis* (Mizukami et al. 1996). So, we alternatively selected a dominant negative mutations technique (Herskowitz 1987) by overexpressing the mutated *OsMADS1*^Olr^ allele in NIP to competitively inhibit the normal function of endogenous wild-type *OsMADS1* of NIP to test whether *OsMADS1*^Olr^ was responsible for the Oat-like rice phenotype. The p*Ubi*::*OsMADS1*^Olr^ vector was constructed and subsequently introduced into the NIP calli by *Agrobacterium*-mediated transformation.

**Fig. 8:**
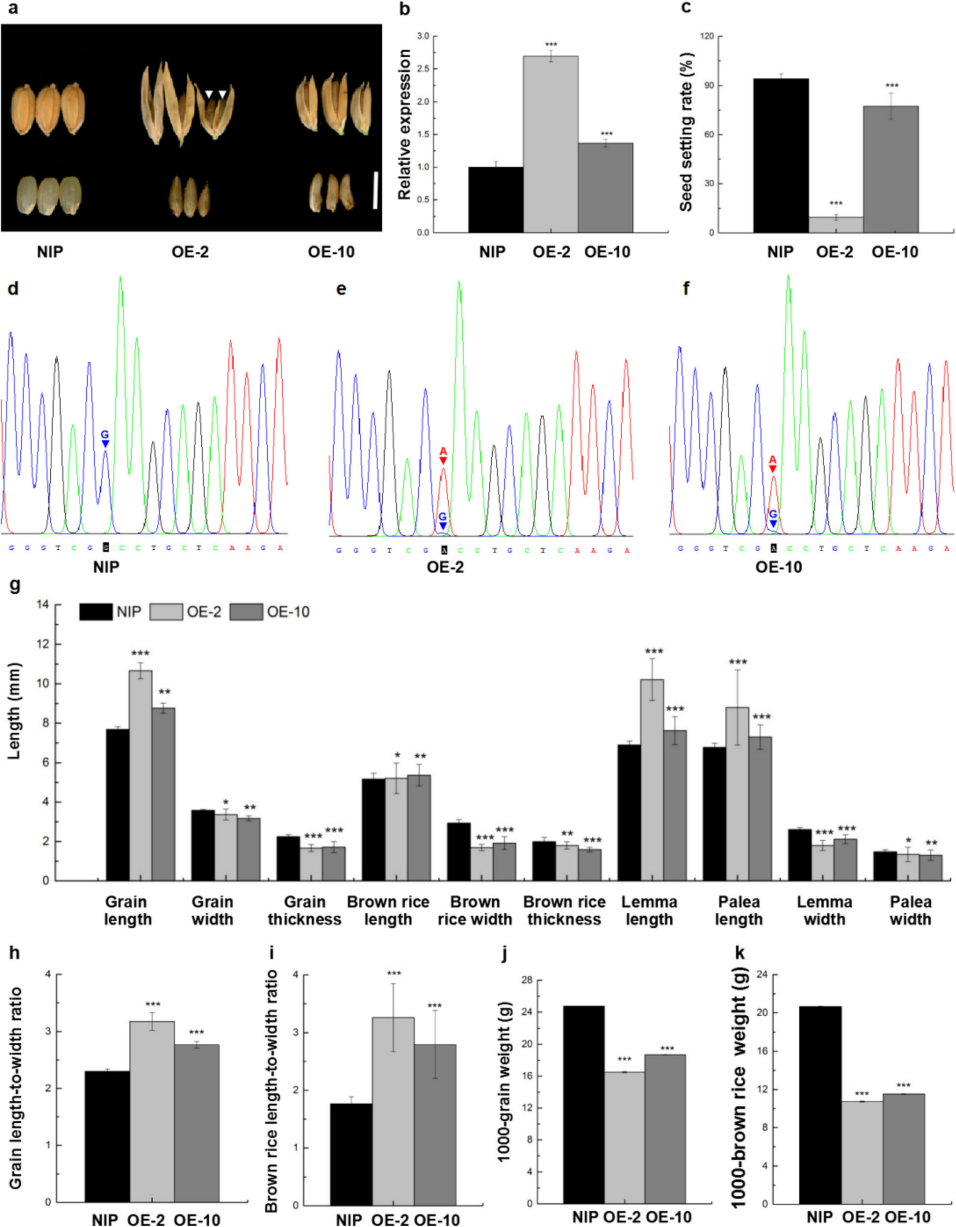
Oat-like rice phenotypes and statistics of *OsMADS1*^Olr^***-***overexpressing transgenic plants. **a** Grain shape and phenotypes of *OsMADS1*^Olr^***-***overexpressing plants (OE-2 and OE-10) with similar Oat-like rice phenotypes in comparison with grains of the wild-type plant (NIP). Arrowheads indicate conjugated twin brown rice. **b** Relative expression of the *OsMADS1* in 12 DAF grains of wild-type and *OsMADS1*^Olr^***-***overexpressing plants. **c** Seed setting rate of wild-type and *OsMADS1*^Olr^***-***overexpressing plants. **d-f** Chromatogram of cDNA fragments spanning the mutation site in *OsMADS1*^Olr^ or the corresponding wild-type site in *OsMADS1* from RT-PCR amplification from NIP (**d**), OE-2 (**e**) and OE-10 (**f**) with a designed *OsMADS1*-RT primer pair (Additional file 1: Table S5). Red arrowheads indicate the signal peak of A at the mutation site in *OsMADS1*^Olr^ cDNA derived from Oat-like rice, and blue arrowheads indicate the signal peak of G at the corresponding wild-type site in *OsMADS1* cDNA derived from NIP. **g** Comparison of grain length, grain width, grain thickness, brown rice length, brown rice width, brown rice thickness, lemma length, palea length, lemma width, and palea width between wild-type and *OsMADS1*^Olr^***-***overexpressing plants. **h-k** Comparison of grain length-to-width ratio (**h**), brown rice length-to-width ratio (**i**), 1000-grain weigh (**j**) and 1000-brown rice weight (**k**) between wild-type and *OsMADS1*^Olr^***-***overexpressing plants. Bar: (**a**) 5 mm. Data presented are mean values ± SDs [*n* = 3 replicates in **b**, **j** and **k**; *n* = 12 replicates in **c**; *n* = 50 in **g-i**.]. Student’s *t*-test: *p < 0.05, **p < 0.01, ***p < 0.001

And nineteen positive primary transgenic plants (T_0_) were obtained and showed normal phenotypes indistinguishable from NIP wild-type plants during the vegetative stage. After PCR verification and qRT-PCR analysis, one severe plant (OE-2) and another weak plant (OE-10) which showed the abnormal grain shape, similar to that of Oat-like rice were selected for detailed phenotypic analysis (Fig. 8a-c). The occasional formation of twin brown rice in one grain of OE-2 was also observed (Fig. 8a, indicated by arrowhead). The lemma length, palea length, grain length and brown rice length of both OE-2 and OE-10 were also significantly longer than those of wild-type (Fig. 8g). Conversely, the grain width, lemma width, palea width, grain thickness, brown rice width and brown rice thickness values were all significantly lower than those of wild-type (Fig. 8g). Thus, OE-2 and OE-10 plants had slender grains and brown rice compared with wild type, as indicated by the higher length-to-width ratio of grain and brown rice (Fig. 8a, h and i). In addition, both the 1000-grain weight and 1000-brown rice weight of OE-2 and OE-10 were extremely significantly decreased compared with wild type (Fig. 8j and k). In addition, OE-2 and OE-10 also had abnormal spikelets with various variations in floral organ numbers and morphologies, which was very similar to the spikelets of Oat-like rice (Additional file 1: Figure S6).

Overall, the OE-2 plant had a more severe Oat-like phenotype than OE-10, including a much lower seed setting rate, which may be ascribed to the possibly stronger expression level of *OsMADS1*^Olr^ in OE-2 than in OE-10 as indicating by much higher expression level of total mutated *OsMADS1*^Olr^ and endogenous wild-type *OsMADS1* in OE-2 than in OE-10 (Fig. 8a-c). However, we were not able to detect the amount of solo *OsMADS1*^Olr^ transcript in neither OE-2 nor OE-10 only by qRT-PCR because it is unlikely to differentiate the mutated *OsMADS1*^Olr^ transcript from the endogenous wild-type *OsMADS1* transcript by this method due to single SNP between *OsMADS1*^Olr^ and *OsMADS1* transcripts. Thus, to further detect the amount of solo *OsMADS1*^Olr^ and the relative ratio of *OsMADS1*^Olr^ transcript to *OsMADS1* transcript in OE-2 and OE-10, we performed RT-PCR amplification with a designed *OsMADS1*-RT primer pair (Additional file 1: Table S5) and subsequently sequenced the cDNA fragments spanning the mutation site in *OsMADS1*^Olr^ or the corresponding wild-type site in *OsMADS1* from NIP (Fig. 8d), OE-2 (Fig. 8e) and OE-10 (Fig. 8f).

And we were surprised to find that the signal peak of mutated A at the mutation site in *OsMADS1*^Olr^ cDNA derived from Oat-like rice was extremely higher than the weak signal peak of wild-type G at the corresponding wild-type site in *OsMADS1* cDNA derived from NIP in both OE-2 and OE-10 (Fig. 8e and f). Considering the weak signal peak of wild-type G at the mutation site but strong signal of the another adjacent G ahead of the mutation site, there also might be the possibility that the weak signal peak may contains the residual background signal of the another adjacent G ahead of the mutation site in OE-2 and OE-10. This result suggested that the overexpressed mutated *OsMADS1*^Olr^ transcript is extremely higher than the endogenous wild-type *OsMADS1* transcript in both OE-2 and OE-10 by using the dominant negative mutations technique. Thus, the Oat-like rice phenotype of OE-2 and OE-10 could be caused by their overexpression of mutated *OsMADS1*^Olr^ and relatively trace expression of endogenous wild-type *OsMADS1*. Taken together, we concluded that the *OsMADS1*^Olr^ was indeed responsible for the Oat-like rice phenotype, including the aberrant grain shape.

### Suppression of *OsMADS1* Expression by RNAi Produced Oat-Like Rice Phenotype

**Fig. 9:**
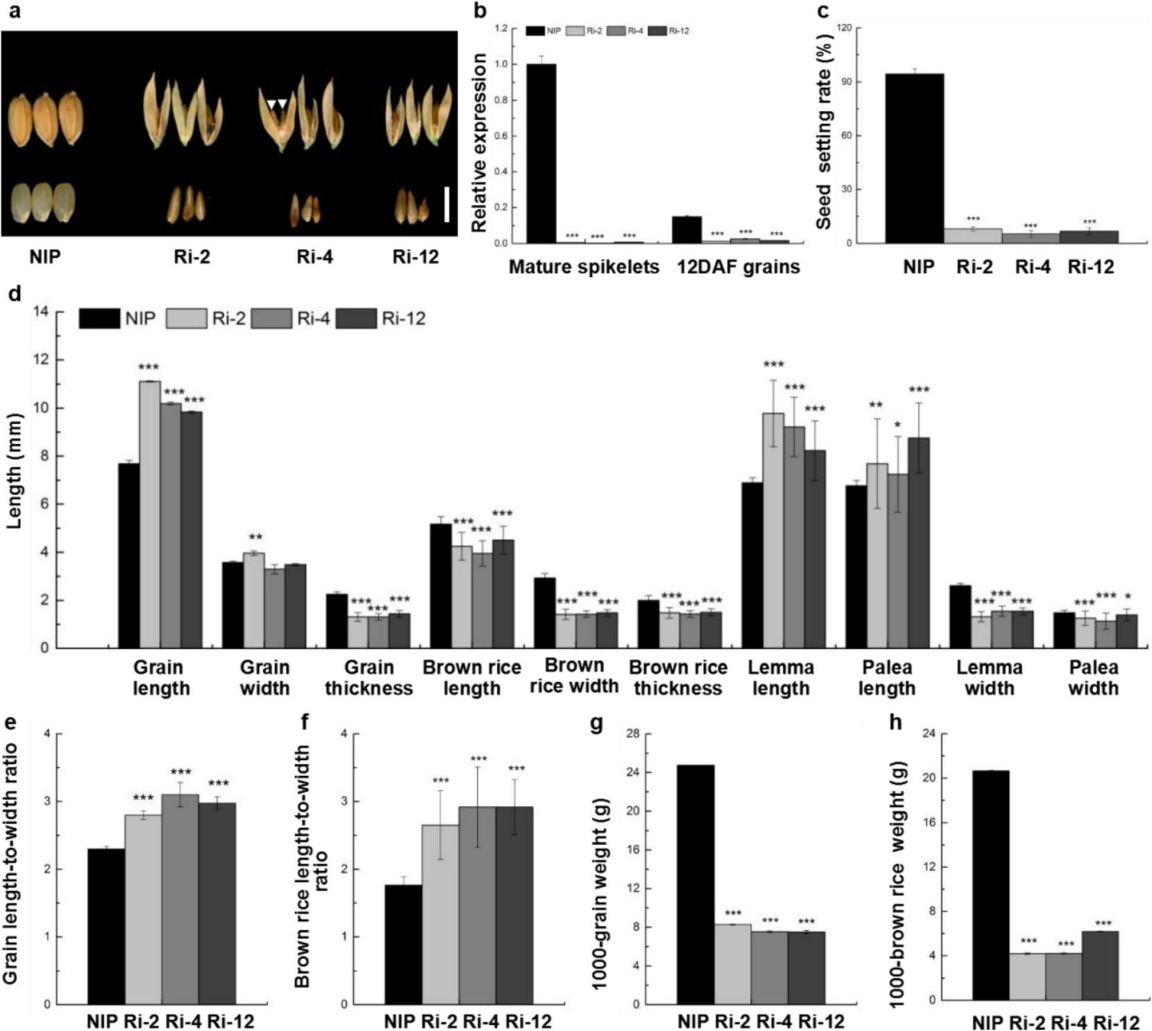
Oat-like rice phenotypes and statistics of *OsMADS1*-RNAi plants. **a** Grain shape and phenotypes of severe *OsMADS1*-RNAi plants (Ri-2, Ri-4 and Ri-12) with similar Oat-like rice phenotypes in comparison with grains of wild-type plant (NIP). Arrowheads indicate conjugated twin brown rice. **b** Relative expression of *OsMADS1* in matures spikelets and 12 DAF grains of wild-type and severe *OsMADS1*-RNAi plants. **c** Seed setting rate of wild-type and severe *OsMADS1*-RNAi plants. **d** Comparison of grain length, grain width, grain thickness, brown rice length, brown rice width, brown rice thickness, lemma length, palea length, lemma width and palea width between wild-type and severe *OsMADS1*-RNAi plants. **e-h** Comparison of grain length-to-width ratio (**e**), brown rice length-to-width ratio (**f**), 1000-grain weigh (**g**) and 1000-brown rice weight (**h**) between wild-type and severe *OsMADS1*-RNAi plants. Bar: (**a**) 5 mm. Data presented are mean values ± SDs [*n* = 3 repeats in **b**, **g** and **h**; *n* = 12 repeats in **c**; *n* = 50 in **d-f**.]. Student’s *t*-test: *p < 0.05, **p < 0.01, ***p < 0.001

To further confirm the roles of the *OsMADS1* gene in regulating grain shape in rice, the p*Ubi*::*OsMADS1*-RNAi vector was constructed and transformed into NIP to suppress the expression of *OsMADS1*. Twenty-three independent T_0_ transformants were obtained, and all the *OsMADS1*-RNAi transgenic plants developed normally during the vegetative stage. Ultimately, three independent transgenic plants (Ri-2, Ri-4 and Ri-12) with the lowest *OsMADS1* expression were chosen for phenotypic analysis (Fig. 9a and b). Compared with NIP, the relative expression of *OsMADS1* in spikelets and grains at 12 DAF was negligible in the three *OsMADS1*-RNAi transgenic lines (Fig. 9b). Additionally, all three *OsMADS1*-RNAi plants had not only abnormal spikelets harboring variations in floral organ numbers and morphologies but also aberrant grains similar to those of Oat-like rice (Additional file 1: Figure S7 and Fig. 9a). Furthermore, the seed setting rate of Ri-2, Ri-4 and Ri-12 plants was extremely reduced compared with wild type but comparable to that of Oat-like rice (Figs. 5j and 9c). In addition, the occasional formation of twin brown rice in one grain of Ri-4 was also observed, as indicated by the white arrowheads (Fig. 9a). Moreover, the lemma length, palea length and grain length of Ri-2, Ri-4 and Ri-12 plants were significantly longer than those of wild type (Fig. 9a and d). Conversely, apart from the increased grain width in Ri-2 plant, the lemma width, palea width, grain thickness, brown rice width, brown rice thickness, 1000-grain weight and 1000-brown rice weight values in Ri-2, Ri-4 and Ri-12 plants were all significantly reduced compared with of wild-type (Fig. 9a, d, g and h). The three *OsMADS1*-RNAi plants had slender grains, slender brown rice, and smaller and lighter brown rice compared with wild type, as indicated by the higher length-to-width ratio of the grain and brown rice, lower 1000-grain weight and 1000-brown rice weight (Fig. 9e-h). In summary, Ri-2, Ri-4 and Ri-12 plants had negligible expression of *OsMADS1* in the spikelets and grains, indicating that they were severe *OsMADS1*-RNAi plants. Additionally, the phenotypes of the three *OsMADS1*-RNAi plants were highly similar to *OsMADS1*^Olr^-overexpressing plants and Oat-like rice, indicating that the severe phenotypes of Oat-like rice in grain shape and floral organs were caused by *OsMADS1*^Olr^.

### Altered Expression of Representative Genes Related to Grain Shape in Oat-Like Rice and Transgenic Plants

**Fig. 10:**
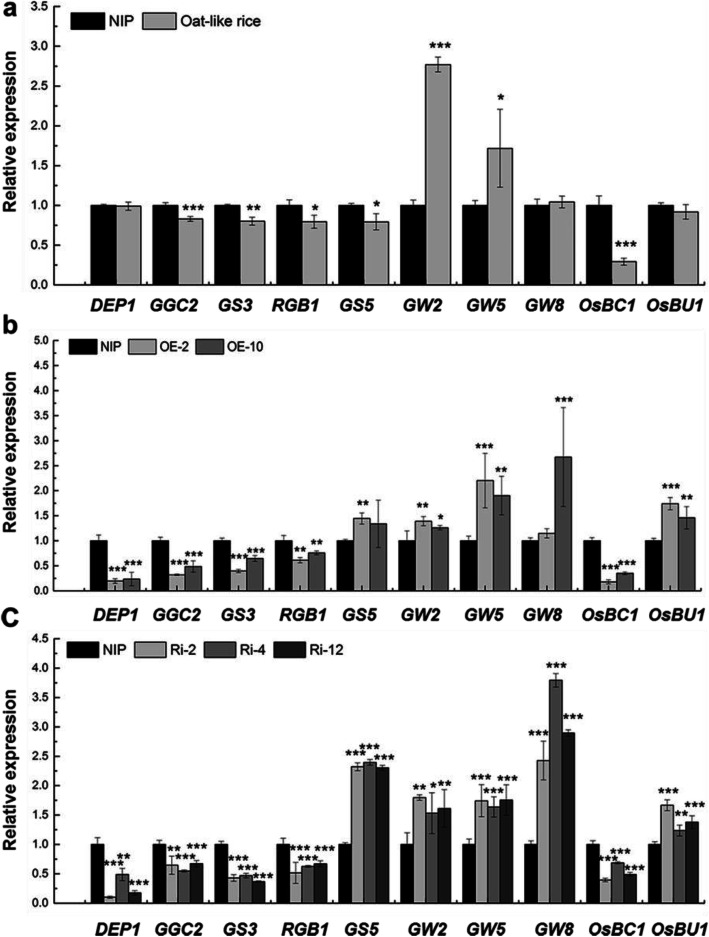
Expression analysis of representative genes related to grain shape in Oat-like rice, *OsMADS1*^Olr^***-***overexpressing and *OsMADS1*-RNAi plants. **a-c** Relative expression of representative genes related to grain shape in NIP and Oat-like rice plants (**a**), Os*MADS1*^Olr^***-***overexpressing plants (**b**) and *OsMADS1*-RNAi plants (**c**) by qRT-PCR analysis. For each sample, total RNA isolated from 12 DAF grains was used for qRT-PCR analysis. The value of *OsActin* mRNA was used as an internal control for data normalization, and the expression levels of genes in NIP were set as 1.0 (**a**-**c**). Values are means ± SDs of three replicates. Student’s *t*-test: *p < 0.05, **p < 0.01, ***p < 0.001

To explore the correlation of *OsMADS1* gene between its function and the regulatory network in controlling grain shape, we analyzed the expression levels of ten representative genes regulating grain shape in Oat-like rice, *OsMADS1*^Olr^-overexpressing and *OsMADS1*-RNAi plants which all showed altered and abnormal grain shape compared with control plants NIP (Figs. 1, 8 and 9). Compared with control plants, in general, we found that each gene had relatively similar expression patterns in Oat-like rice, *OsMADS1*^Olr^-overexpressing and *OsMADS1*-RNAi plants in grains at 12 DAF by qRT-PCR assays. The results indicated that the expression levels of *DEP1*, *GGC2* and *RGB1* were significantly decreased in Oat-like rice, *OsMADS1*^Olr^-overexpressing and *OsMADS1*-RNAi plants, except that *DEP1* showed no significant difference in expression levels between Oat-like rice and NIP. And transcript levels of *GS3* were also reduced significantly, which is consistent with the increased grain length of Oat-like rice, *OsMADS1*^Olr^-overexpressing and *OsMADS1*-RNAi plants. Additionally, although *GS5* and *GW8* showed decreased or increased transcript levels respectively in Oat-like rice, transcript levels of both *GS5* and *GW8* were elevated in *OsMADS1*^Olr^-overexpressing and *OsMADS1*-RNAi plants. Furthermore, both *GW2* and *GW5* showed significantly upregulated expression levels in Oat-like rice, *OsMADS1*^Olr^-overexpressing and *OsMADS1*-RNAi plants, which is consistent with the decreased lemma width and palea width of these plants. Additionally, transcript levels of *OsBC1* were significantly downregulated in Oat-like rice, *OsMADS1*^Olr^-overexpressing and *OsMADS1*-RNAi plants. Additionally, expression levels of *OsBU1* was decreased in Oat-like rice but significantly increased in *OsMADS1*^Olr^-overexpressing and *OsMADS1*-RNAi plants (Fig. 10a-c). These results indicated that the differentially-expressed *DEP1*, *GGC2*, *RGB1*, *GS3*, *GW2*, *GW5*, *GW8* and *OsBC1* were shared in Oat-like rice and the transgenic lines, suggesting that *OsMADS1* regulates grain shape possibly by affecting the expression levels of these genes.

### *OsMADS1* May Has a Direct Role in Seed Development

The *OsMADS1*^Olr^-overexpression and *OsMADS1*-RNAi plants showed abnormal grain shape similar to that of Oat-like rice, which indicated that *OsMADS1*^Olr^ is responsible for phenotypes in grain shape of Oat-like rice. Nevertheless, it is unlikely to exclude the secondary effect brought by the aberrant lemma and palea development to the final grain shape of Oat-like rice. To solve the problem whether *OsMADS1* has a direct role in seed development independent of lemma and palea development, we used seed-specific *OsMADS1*-RNAi technique to specifically inhibit the expression of *OsMADS1* during grain development.

We cloned the promoter of *OsTip3* gene, which is specifically expressed in grains including embryo and endosperm, but not in vegetative tissues of rice plants such as root and shoot (Takahashi et al. 2004). The public microarray data from ePlant Rice database (http://bar.utoronto.ca/eplant_rice/) show the *OsTip3* gene is only expressed in seed (starting from seed S2 stage) but not in inflorescence (Additional file 1: Figure S8b) (Itoh et al. 2005; Waese et al. 2017). And the RiceXPro results (https://ricexpro.dna.affrc.go.jp/category-select.php) confirmed the expression pattern of *OsTip3* and also indicated that its expression level is much higher than that of *OsMADS1* during seed development in general (Additional file 1: Figure S8c and d) (Sato et al. 2013). Moreover, expression analysis of both *OsTip3* and *OsMADS1* in representative stages of inflorescences, spikelets and grains by our transcriptome analysis between Oat-like rice and NIP also supported the above results (data not shown). Thus, these results suggested that *OsTip3* promoter (p*OsTip3*) could be used as seed-specific promoter to silence *OsMADS1* gene.

The p*OsTip3*::*OsMADS1*-RNAi vector was constructed and subsequently transformed into NIP. Thirty-one positive primary transgenic plants (T_0_) were subsequently obtained and all displayed undifferentiated phenotypes from wild-type plants during the vegetative and reproductive stages. As shown in Additional file 1: Figure S9, T_0_ plants eventually produced normal and mature spikelets with closed lemma and palea. However, mature grains of these plants showed mild defect with a narrow crack in either side or both sides of a grain, and expression levels of *OsMADS1* in the developing grains of these grains were extremely (T3-Ri-3 (T_0_), T3-Ri-4 (T_0_) and T3-Ri-7 (T_0_)) or significantly (T3-Ri-6 (T_0_)) reduced (Additional file 1: Figure S10).

**Fig. 11:**
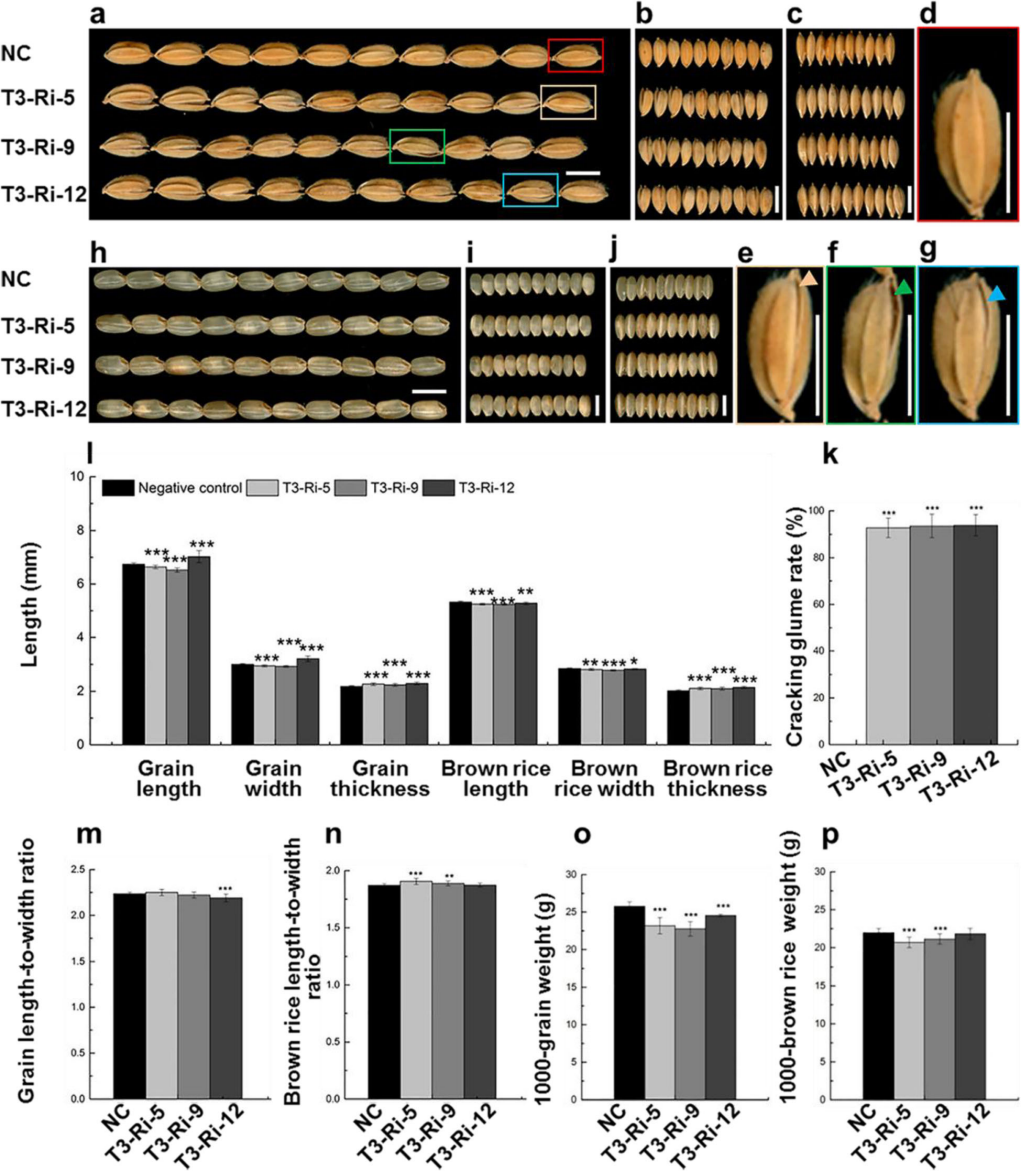
Seed-specific interfering phenotypes and statistics of p*OsTip3*::*OsMADS1*-RNAi lines. **a**-**g** Grain shape and phenotypes of T_2_ p*OsTip3*::*OsMADS1*-RNAi lines (T3-Ri-5, T3-Ri-9 and T3-Ri-12) in comparison with grains of the negative control (NC) line. **d**-**g** Enlarged view of representative grains of negative control line (**d**), T3-Ri-5 (**e**), T3-Ri-9 (**f**) and T3-Ri-12 (**g**) lines, which corresponding to red (**d**), orange (**e**), green (**f**) and blue (**g**) rectangles in **a**, respectively. Arrowhead indicates a crack formed by incompletely closed glume in one side of a grain (**e**-**g**). **h**-**j** Shape and phenotypes of brown rice of p*OsTip3*::*OsMADS1*-RNAi lines (T3-Ri-5, T3-Ri-9 and T3-Ri-12) in comparison with that of the negative control line. **k** Comparison of cracking glume rate between p*OsTip3*:*:OsMADS1*-RNAi lines and the negative control line. **l** Comparison of grain length, grain width, grain thickness, brown rice length, brown rice width and brown rice thickness between p*OsTip3*::*OsMADS1*-RNAi lines and the negative control line. **m**-**p** Comparison of grain length-to-width ratio (**m**), brown rice length-to-width ratio (**n**), 1000-grain weigh (**o**) and 1000-brown rice weight (**p**) between p*OsTip3*::*OsMADS1*-RNAi lines and negative control line. Bars: (**a**-**j**) 5 mm. Data presented are mean values ± SDs [*n* = 27 plants for each line in **k**; *n* = 24 plants for each line in **l**-**p**.]. Student’s t-test: *p < 0.05, **p < 0.01, ***p < 0.001

To obtain more accurate result, we performed further detailed statistical analysis by using another three homozygous p*OsTip3*::*OsMADS1*-RNAi lines. Plants of T_2_ lines (T3-Ri-5, T3-Ri-9 and T3-Ri-12) also developed normal and mature spikelets with closed lemma and palea. However, although T3-Ri-5, T3-Ri-9 and T3-Ri-12 exhibited seemingly similar grain shape with the negative control line, the mild defect of a narrow crack in either side (63.91 ~ 69.00%) or both sides (20.36 ~ 23.67%) of a grain can differentiate them from the negative control line. Nevertheless, the grain shape of T3-Ri-5, T3-Ri-9 and T3-Ri-12 was apparently different from that of *OsMADS1*^Olr^-overexpression and *OsMADS1*-RNAi plants with open lemma and palea which affected inner brown rice (Figs. 8a, 9a, 11a, e-g, Additional file 1: Figure S11).

Although T3-Ri-5, T3-Ri-9 and T3-Ri-12 didn’t exhibit consistent difference in the length and width of grain as compared with negative control (Fig. 11a-l), the length and width of brown rice in these lines were significantly decreased (Fig. 11h, i, and l). However, *OsMADS1*^Olr^-overexpression and *OsMADS1*-RNAi plants exhibited more decrease in brown rice width (Figs. 8g, 9d). Besides, the three lines showed relatively comparable grain length-to-width ratio and brown rice length-to-width ratio to that of the negative control line. It is different from *OsMADS1*^Olr^-overexpression and *OsMADS1*-RNAi plants with slender grains and brown rice as partly indicating by significantly increased grain length-to-width ratio and brown rice length-to-width ratio. In addition, unlike the remarkably decreased 1000-grain and 1000-brown rice weight of both *OsMADS1*^Olr^-overexpression and *OsMADS1*-RNAi plants, the three lines only exhibited slight or mild decrease in both 1000-grain and 1000-brown rice weight compared with the negative control line (Figs. 8a and h-k, 9a and e-h, 11a-j and l-p).

Interestingly, the thickness of grain and brown rice in these lines were both significantly increased (increased by 2.73 ~ 5.21%, 4.02 ~ 6.29%, respectively) (Fig. 11c, j and l), which are different from that of *OsMADS1*^Olr^-overexpressing and *OsMADS1*-RNAi plants (Figs. 8 a and g, 9 a and d). Besides, more than 90% grains of these lines displayed mild defect with a narrow crack in either side or both sides of a grain (Fig. 11a,e-g and k, Additional file 1: Figure S11), possibly resulting from the push of inner brown rice which are thicker.

Compared with *OsMADS1*^Olr^-overexpressing and *OsMADS1*-RNAi plants, seed-specific *OsMADS1*-RNAi lines produced another kind of grain shape with mild defect with a narrow crack in either side or both sides of a grain, our results indicated that *OsMADS1* may directly regulate seed development, independent of lemma and palea development. Therefore, our results also indicated that the dramatic slender grain phenotypes of Oat-like rice, *OsMADS1*^Olr^-overexpressing, and *OsMADS1*-RNAi plants are majorly caused by the abnormal lemma and palea development.

## Discussion

In this study, we characterized Oat-like rice with severe developmental defects in floral organs and grains. The morphological characteristics of Oat-like rice included the open hull, extremely-elongated leafy lemma-palea, and brown rice with various shapes (Fig. 1). By map-based cloning, we revealed that the coding region of *OsMADS1* had a single point mutation in Oat-like rice (Fig. 6). Previous studies have reported some allelic mutants of *OsMADS1* gene harbors different mutation sites in the gene, but these mutation sites differ from *OsMADS1*^Olr^ in Oat-like rice. Although *lhs1* and *nsr* carry one of the same mutation site as *OsMADS1*^Olr^ in Oat-like rice, they also have other mutation sites in *OsMADS1* (Kinoshita et al. 1976; Chen and Zhang 1980; Jeon et al. 2000; Chen et al. 2006). Additionally, the other allelic mutants or variants of the *OsMADS1* gene, *such as NF1019*, *ND2920*, *NE3043* and *NG778* (Agrawal et al. 2005), *osmads1-z* (Gao et al. 2010; Hu et al. 2015), *ohms1* (Sun et al. 2015), *cyc15* (Zhang et al. 2018), SLG-1 (Yu et al. 2018) and L-204 (Liu et al. 2018), did not carry the mutation site of *OsMADS1*^Olr^. Most studies of these mutants focused on the effects of *OsMADS1* on spikelet development (Kinoshita et al. 1976; Chen and Zhang 1980; Jeon et al. 2000; Agrawal et al. 2005; Chen et al. 2006; Gao et al. 2010; Hu et al. 2015; Sun et al. 2015; Zhang et al. 2018). However, *OsMADS1*^*lgy3*^ was reported to mediate grain size and resulted in long-regular grains and higher yield (Liu et al. 2018). All together, we considered Oat-like rice to be a spontaneous mutant for exploring the mechanisms of grain development. The mutation site was associated with the Oat-like rice phenotype and conserved in MADS-box family members (Additional file 1: Figures S4 and S5). These results suggest that Gly, the 27th amino acid in the MADS domain of OsMADS1, is important for the function of *OsMADS1* in regulating grain shape.

Previous studies have shown that *OsMADS1* is mainly expressed in the floral meristems, the panicles and also during grain development (Chung et al.1994; Arora et al. 2007; Liu et al. 2018; Zhang et al. 2018). Our qRT-PCR and GUS staining results showed that *OsMADS1* was also highly expressed in the early development of grains. Particularly, its expression in grain epidermis, embryo, endosperm, and the aleurone layer was visualized herein in our GUS staining assay (Fig. 7). These results indicated that the *OsMADS1* gene may affect the regulation of grain development. The *OsMADS1*^Olr^-overexpressing and *OsMADS1*-RNAi plants showed morphological variation resembling the aberrant grain shape of Oat-like rice (Figs. 8 and 9), confirming that the phenotype of Oat-like rice is caused by *OsMADS1*^Olr^ and that *OsMADS1* plays a critical role in grain shape development.

In this study, we showed that the mutated *OsMADS1*^Olr^ that caused the Oat-like rice phenotype is a recessive allele. However, it is seemingly paradoxical but also very interesting that overexpression of the *OsMADS1*^Olr^ recessive allele in NIP by a dominant negative mutations technique (Herskowitz 1987) resulted in a dominant Oat-like rice phenotype in the T_0_ *OsMADS1*^Olr^-overexpressing transgenetic plants (OE-2 and OE-10). And Jeon et al. (2000) also reported that ectopic expression of the mutated *OsMADS1*^*lhs1*^ allele harboring two mutation sites in the MADS domain including one of the same mutation site as *OsMADS1*^Olr^ caused phenotypic alterations of spikelets in transgenic panicles, which resembles the *lhs1* mutant. In addition, over-expression of the mutated allele of the *AG *in *Arabidopsis* lacking the C-terminal region also caused *ag*-like phenotypes in the transgenetic plants with abnormal flowers and floral organs resembling the *ag* mutant (Mizukami et al. 1996). Actually, before the widely use of RNAi and gene-editing techniques, the dominant negative mutations as used in the above cases was an common and effective technique for the functional analysis of a target gene, especially for the genes whose encoding proteins are parts of a complex, because overexpressed mutated protein can competitively inhibit the normal function of the coexisting endogenous one (Herskowitz 1987; Goff et al. 1991; Mizukami et al. 1996; Jeon et al. 2000).

In contrast to the dominant Oat-like rice phenotype of the OE-2 and OE-10 plants overexpressing the *OsMADS1*^Olr^ recessive allele in NIP which harbors two copies of endogenous wild-type *OsMADS1*, the heterozygous *OsMADS1* / *OsMADS1*^Olr^ F_1_ plants that derived from a cross between Oat-like rice and NIP showed a normal grain shape phenotype similar to NIP which suggested that the Oat-like rice phenotype and *OsMADS1*^Olr^ allele are recessive in *OsMADS1* / *OsMADS1*^Olr^ F_1_ plants. This seeming paradox and discrepancy could be explained by the different ratio of mutated *OsMADS1*^Olr^ transcripts to wild-type *OsMADS1* transcripts between *OsMADS1* / *OsMADS1*^Olr^ F_1_ plants and T_0_ *OsMADS1*^Olr^-overexpressing plants. In *OsMADS1* / *OsMADS1*^Olr^ F_1_ plants, the amount of mutated *OsMADS1*^Olr^ transcript and OsMADS1^Olr^ protein should theoretically be corresponding equal to that of the wild-type *OsMADS1* transcript and OsMADS1 protein, respectively. As a result, this equal amount of mutated OsMADS1^Olr^ protein may be not sufficient to competitively inhibit the normal function of wild-type OsMADS1 protein to subsequently cause the Oat-like rice phenotype, which led to a recessive effect of *OsMADS1*^Olr^ in the *OsMADS1* / *OsMADS1*^Olr^ F_1_ plants. However, in both OE-2 and OE-10 plants, overexpression of *OsMADS1*^Olr^ resulted in the extremely higher amount of mutated *OsMADS1*^Olr^ transcript than the trace *OsMADS1* transcript even if considering the effect of possible background signal of the another adjacent G ahead of the mutation site (Fig. 8d-f). As a result, the amount of mutated OsMADS1^Olr^ protein is expected to be much more than the wild-type OsMADS1 protein.

Previous studies reported that the transcription factor OsMADS1 interacts with some of the G-protein βγ subunits like OsRGB1, OsRGG1, OsRGG2, DEP1 and GS3 to form a complex through its K-box domain to enhance OsMADS1 transcriptional activity, thereby regulating grain shape and grain yield (Liu et al. 2018). In OsMADS1^Olr^, the one amino acid substitution from conserved glycine (Gly) to aspartic acid (Asp) is located in the MADS domain (Fig. 6d), but the K-box domain is unaffected and expected to be normal. Therefore, in OE-2 and OE-10 plants, the mutated OsMADS1^Olr^ protein is expected to be able to interact with these G-protein βγ subunits and other cofactors just like the wild-type OsMADS1 protein to form a complex. Furthermore, the amount of formed OsMADS1^Olr^-G-protein βγ subunits-cofactors complex should theoretically be much more than the OsMADS1-G-protein βγ subunits-cofactors complex due to competitive binding effect.

However, the OsMADS1^Olr^-G-protein βγ subunits-cofactors complex may be hard or unable to bind to the target genes of OsMADS1^Olr^ to promote transcription of these target genes. Because the DNA-binding ability of OsMADS1^Olr^ protein may be compromised, which can be attributed to the amino acid substitution from conserved Gly to Asp in the MADS domain of OsMADS1^Olr^. This assumption is consistent with the studies of Pellegrini et al. (1995). The 27th wild-type amino acid (Gly) in OsMADS1 is also conserved (equivalent Gly 167) in the MADS-box domain of the human MADS-box transcription factor, SRF. The X-ray crystal structure of the SRF core polypeptide in a specific-recognition DNA complex showed that the conserved Gly 167 is located in the predominant element of the DNA-binding motif of the MADS-box domain, the coiled coil formed αI-helix. The Gly 167 amino acid is essential for the close approach of the αI-helix and MADS-motif coiled coil to the SRF target DNA sites, thereby promoting binding and activation of SRF transcription factor to its target genes (Pellegrini et al. 1995). On the other hand, the trace OsMADS1-G-protein βγ subunits-cofactors complex maybe insufficient to promote transcription of the target genes of OsMADS1 to further regulate the normal development of spikelet and grain, and subsequently caused the Oat-like rice phenotype, which caused a dominant negative effect of *OsMADS1*^Olr^ in OE-2 and OE-10 plants.

In rice, grain shape is closely related to spikelet development because the final grain shape is coordinately controlled by cell proliferation and cell expansion in the spikelet hull, which consists of a lemma and a palea (Li et al. 2018). And previous studies indicated that *OsMADS1* regulates spikelet development partly by specification of the lemma and palea and acts as an inhibitor of overdevelopment of the lemma and palea (Jeon et al. 2000; Prasad et al. 2001; Chen et al. 2006; Wang et al. 2017). Furthermore, recent studies indicated that *OsMADS1* not only regulates spikelet development, but also plays important roles in regulating grain shape (Liu et al. 2018; Sun et al. 2018; Yu et al. 2018; Wang et al. 2019).

In Oat-like rice, *OsMADS1*^Olr^-overexpressing and *OsMADS1*-RNAi transgenic plants, the normal function of *OsMADS1* was theoretically affected or compromised in both spikelets and grains. Therefore, the dramatic grain phenotypes of these plants should contain a secondary effect brought by the aberrant lemma and palea development caused by the affected or compromised function of *OsMADS1*. However, whether the dramatic grain phenotypes of these plants also contain the effect of aberrant grain development resulted from affected or compromised function of *OsMADS1* in grains, and if *OsMADS1* has a direct regulation role in grain development after pollination or *OsMADS1* only indirectly regulates grain development by controlling the lemma and palea development before pollination? To answer this question and solve the problem that the secondary effect brought by the aberrant lemma and palea development to the final grain shape was unable to be excluded in *OsMADS1*^Olr^-overexpressing and *OsMADS1*-RNAi plants, the seed-specific *OsMADS1*-RNAi technique is an ideal alternative. And previous studies also reported use of seed-specific RNAi technique to specifically inhibit the expression of a target gene in grains to explore its specific function in regulating seed development or grain quality (Ali et al. 2013; Zhang et al. 2017).

Compared with seed-specific *OsMADS1*-RNAi plants, Oat-like rice, *OsMADS1*^Olr^-overexpressing and *OsMADS1*-RNAi plants displayed much more severe abnormal grain phenotypes with open lemma and palea. And seed-specific *OsMADS1*-RNAi lines only exhibited mild defect in grains with a narrow crack in either side or both sides of a grain. And expression of *OsMADS1* was extremely or significantly reduced in developing grains of T_0_ seed-specific *OsMADS1*-RNAi plant which showed similar phenotype to T_2_ seed-specific *OsMADS1*-RNAi lines. These results indicated that OsMADS1 may indeed has a direct role, which is independent of lemma and palea development, in seed development. And the abnormal lemma and palea development is the major reason for abnormal phenotypes of Oat-like rice, *OsMADS1*^Olr^-overexpressing, and *OsMADS1*-silenced plants in our case.

Interestingly, the three seed-specific *OsMADS1*-RNAi lines showed significantly thicker grain and brown rice, which are opposite to that of *OsMADS1*^Olr^-overexpressing, and *OsMADS1*-silenced plants. Grain thickness usually reflects and is related to grain filling. Thus this result suggests that the grain filling of the seed-specific *OsMADS1*-RNAi lines might be affected. And the narrow crack and cracks in grains of these lines is likely to be resulted from the push of inner thicker brown rice, which is possibly resulted from disordered grain filling. It requires further investigations to elucidate how *OsMADS1* mediates the thickness of grain and grain filling.

The previous research has reported that *DEP1*, *GGC2* and *RGB1* are positive regulatory genes, while *GS3* is a negative regulatory gene for grain length (Sun et al. 2018). In our study, we found that the expression of *DEP1*, *GS3*, *GGC2* and *RGB1* were changed significantly in Oat-like rice, *OsMADS1*^Olr^-overexpressing plants and *OsMADS1*-RNAi transgenic plants, except that expression of *DEP1* did not changed in Oat-like rice (Fig. 10). Previous studies have shown that *DEP1* might be a target gene of OsMADS1 (Khanday et al. 2013; Hu et al. 2015; Khanday et al. 2016;) and DEP1 and GS3 could interact with OsMADS1 to control grain size (Liu et al. 2018). Our results implied that *OsMADS1* may coordinate with these genes to control grain shape. Of course, further investigations are required to explore how *OsMADS1* interacts with *DEP1*, *GS3*, *GGC2* and *RGB1* to regulate grain shape in the future. In addition, *GW2* and *GW5* negatively regulate genes involved in grain width and weight. The loss of *GW2* or *GW5* function enhances grain width, weight and yield (Song et al. 2007; Duan et al. 2017; Liu et al. 2017). The expression levels of *GW2* and *GW5* genes were significantly upregulated in Oat-like rice, *OsMADS1*^Olr^-overexpressing plants and *OsMADS1*-RNAi transgenic plants (Fig. 10), which may explain the phenotypes in these plants of narrow lemma width, palea width, brown rice and low grain weight or brown rice weight on the whole. In addition, *OsBC1* is a positive regulator of the BR response and grain size. It is reported that the grains of *OsBC1*-dsRNAi plants are smaller than those of control plants (Tanaka et al. 2009; Jang et al. 2017), which may explain the reason why Oat-like rice, *OsMADS1*^Olr^*-*overexpressing plants and *OsMADS1*-RNAi plants had smaller brown rice (Fig. 10), because *OsBC1* is significantly down-regulated in these plants. Therefore, the *OsMADS1* gene influenced the expression of these genes regulating grain shape and played a vital role in regulation of grain shape.

Many factors influence the seed setting rate, including the pollen and embryo sac. And the pollen and embryo sac fertility contributed almost equally to spikelet fertility (Song et al. 2005)*.* Our results indicated that the seed setting rate of Oat-like rice was much lower than that of NIP (Fig. 5j). Additionally, we found that Oat-like rice had aberrant floral organs (Additional file 1: Figure S3 and Table S2). Furthermore, there was no significant difference in pollen vitality and germination ratio between NIP and Oat-like rice, but Oat-like rice had defects in some of the carpels and embryo sacs (Fig. 5a-i). Many previous studies have reported that the *OsMADS1* gene mainly functions in floral identification (Jeon et al. 2000; Agrawal et al. 2005; Prasad et al. 2005; Chen et al. 2006; Arora et al. 2007; Hu et al. 2015; Sun et al. 2015; Zhang et al. 2018). In our study*, OsMADS1* was expressed in pistils but not in stamens, implying that *OsMADS1* is functional in pistils but nonfunctional in stamens (Fig. 7a). Indeed, this finding is consistent with the normal morphologies of stamens, undifferentiated pollen vitality and germination ratio but some of the defective carpels and embryo sacs in Oat-like rice (Fig. 5a-i). Therefore, we speculate that the variation of floral organs and the abnormality of some of the embryo sacs (Additional file 1: Figure S3 and Fig. 5f-i) might be partly related to the low seed setting rate in Oat-like rice.

In general, our findings indicate that the Oat-like rice with various shapes of brown rice represents a spontaneous mutant for studying the function of *OsMADS1* in grain development. Additionally, *OsMADS1* plays a crucial role in regulating grain shape. Further investigations are still required to explore the function of the *OsMADS1* gene in regulating grain shape.

## Conclusions

In this work, we found that the aberrant grain shape of Oat-like rice was caused by a novel allele *OsMADS1*^Olr^. The Oat-like rice phenotype of *OsMADS1*^Olr^-overexpressing and *OsMADS1*-RNAi plants confirmed that *OsMADS1*^Olr^ was responsible for the observed phenotypes of Oat-like rice. The *OsMADS1* gene was highly expressed during the early stages of grain development. Mutation or disruption of *OsMADS1* led to the altered expression levels of representative genes mediating grain shape. Seed-specific *OsMADS1*-RNAi plants verified that *OsMADS1* may play a direct regulation role in grain development. Taken together, the *OsMADS1* gene plays a vital role in regulating grain shape in rice.

## Methods

### Plant Materials and Growth Conditions

The Oat-like rice used in this study is a spontaneous mutant, which was originally discovered in the paddy field in 2001 and might originate from natural variation. Since its original wild type is unknown, the *japonica* cv. Nipponbare (NIP) was used as a control in the phenotype and sequencing analyses, and as a transgenic recipient and wild-type control in transgenic experiments. Oat-like rice and NIP were used as the maternal and paternal plant, respectively, to generate a F_2_ mapping population for mapping the *OsMADS1*^Olr^ gene. Rice plants were grown under natural growing conditions during natural growing seasons in Shifang and Chengdu, Sichuan province of China, and during dry seasons in Lingshui, Hainan province of China.

### Map-Based Cloning of the *OsMADS1*^Olr^ Gene

A F_2_ mapping population was generated by crossing Oat-like rice with NIP. For preliminary mapping of the *OsMADS1*^Olr^ gene, ten individual plants with Oat-like rice phenotype and ten individual plants with normal phenotype were respectively selected to prepare the two segregating DNA bulks. The DNA bulks were subjected to BSA to screen the linked markers of the *OsMADS1*^Olr^ gene by using 175 InDel molecular makers. The genotypes of another 152 individual plants with Oat-like phenotypes in the F_2_ mapping population were analyzed for preliminary mapping of the *OsMADS1*^Olr^ gene. For fine mapping of the *OsMADS1*^Olr^ gene, 1450 individual plants with Oat-like phenotypes in the F_2_ mapping population were selected for genotyping to narrow down the interval of the *OsMADS1*^Olr^ gene. The SSR (Simple Sequence Repeat) and InDel molecular makers used and developed for fine mapping of *OsMADS1*^Olr^ gene are listed in Additional file 1: Table S4. For cloning the *OsMADS1* or *OsMADS1*^Olr^ gene and sequence alignment between NIP and Oat-like rice, the genomic DNA and cDNA of the *OsMADS1* or *OsMADS1*^Olr^ gene from NIP and Oat-like rice were amplified and sequenced, respectively.

### Vector Construction and Rice Transformation

For construction of the *OsMADS1*^Olr^*-*overexpression vector, the *OsMADS1*^Olr^ coding sequence was amplified from Oat-like rice and inserted into the binary vector pCUbi1390 (Peng et al. 2009) at the BamHI site and the KpnI site. The new transformation vector was named p*Ubi*::*OsMADS1*^Olr^.

For construction of the *OsMADS1-*RNAi vector, a 423-bp cDNA fragment of *OsMADS1* was amplified from NIP and used to construct the p*Ubi*::*OsMADS1*-RNAi vector by inserting the cDNA fragment into the left and right MCS (Multiple Cloning Sites) of the pLHRNAi vector (Li et al. 2013) in sense and antisense orientations, respectively. The new vector was named p*Ubi*::*OsMADS1*-RNAi.

For construction of the seed-specific *OsMADS1-*RNAi vector driven by the seed-specific promoter p*OsTip3* in rice, a 1211-bp promoter of *OsTip3* gene was amplified from the genomic DNA of NIP, and subsequently inserted into the HindIII and KpnI site of the pLHRNAi vector (Li et al. 2013). The new vector was named p*OsTip3*-RNAi. Then, a 423-bp cDNA fragment of *OsMADS1* was amplified from NIP and subsequently inserted into the left and right MCS of p*OsTip3*-RNAi. The new vector was named p*OsTip3*::*OsMADS1*-RNAi.

For construction of the *GUS* expressing vector, a 2147-bp promoter of *OsMADS1* was amplified from the genomic DNA of NIP, and subsequently inserted into the BamHI and NcoI site of the binary vector pCAMBIA1305.1. The new transformation vectors were named p*OsMADS1*::*GUS*.

The p*Ubi*::*OsMADS1*^Olr^, p*Ubi*::*OsMADS1*-RNAi, p*OsTip3*::*OsMADS1*-RNAi and p*OsMADS1*::*GUS* plasmids were introduced into the NIP calli, respectively, by *Agrobacterium tumefaciens* (EHA105)-mediated transformation (Hiei et al. 1994). The schematic diagrams of the transgenic vectors are shown in Additional file 1: Fig. S12, and the corresponding primers developed for vector construction are listed in Additional file 1: Table S7.

### Microscope Observation and Histocytological Analysis

Young inflorescences and spikelets were collected to observe and compare the morphologies and developmental courses of the spikelet, lemma and palea between NIP and Oat-like rice under a scanning electron microscope (SEM) based on the method of Li et al. (2013). Anthers harvested from mature spikelets shortly before anthesis were stained with 1% I_2_-KI solution to analyze the pollen viability of NIP and Oat-like rice. Pollens from dissected anthers were cultivated with medium (20% sucrose, 10% PEG 4000, 3 mmol/L Ca (NO3)_2_, 40 mg/L H_3_BO_3_, and 3.0 mg/L VB1) at 28 ± 1 °C for 60 min in dark. The samples were then observed under Nikon light microscope (Nikon, ECLIPSE E200, made in China) and photographed to calculate the pollen germination rate. Samples of spikelets, paleae and pistils from NIP and Oat-like rice were collected and fixed in FAA solution, followed by a series of dehydration and infiltration steps before embedding in paraplast. After sectioning, the sections were dewaxed with xylene, rehydrated, stained with 1% toluidine blue and observed under a Nikon light microscope (Nikon, ECLIPSE E200, made in China). For observation and analyses of the morphologies of spikelets and floral organs from NIP, Oat-like rice, *OsMADS1*^Olr^-overexpression plants and *OsMADS1*-RNAi plants, spikelets were harvested shortly before anthesis and observed under a stereoscopic microscope (Nikon, SMZ745T, made in China).

### RNA Extraction, qRT-PCR, RT-PCR and Sequencing Analysis

Various floral organs including lemmas, paleae, stamens and pistils of NIP and Oat-like rice, and grains of NIP, Oat-like rice, *OsMADS1*^Olr^*-*overexpression and *OsMADS1*-RNAi plants were collected from field-grown rice plants and subsequently stored at − 80 °C after freezing in liquid nitrogen. Total RNA was extracted from all samples using the RNA prep pure Plant kit (TransGen Biotech, Beijing, China). First-strand cDNAs were synthesized from 2 μg total RNA using the TransScript All-in-One First-Strand cDNA Synthesis SuperMix for qPCR (One-Step gDNA Removal) (TransGen Biotech, Beijing, China). The qRT-PCR was performed using SYBR Green Mix (CWBIO, Beijing, China) and run on a CFX Connect™ Real-Time PCR Detection System (Bio-Rad Laboratories, Inc. USA). The *OsActin* gene in rice was used as an endogenous control. The primers used in the qRT-PCR analysis are listed in Additional file 1: Table S6. To differentiate between the mutated *OsMADS1*^Olr^ and wild-type *OsMADS1* transcripts by a combined RT-PCR amplification and sequencing method, cDNA fragments spanning the mutation site in *OsMADS1*^Olr^ or the corresponding wild-type site in *OsMADS1* were amplified respectively from NIP, OE-2 and OE-10 plants with a designed *OsMADS1*-RT primer pair (Additional file 1: Table S5), and sequenced subsequently.

## Supplementary information

**Additional file 1: Figure S1.** Morphologies of NIP and Oat-like rice at maturation stage. **Figure S2.** Grain shape (**a**) and grain length (**b**) of 23 representative rice varieties or accessions from 15 countries in the world in comparison with that of Oat-like rice. Bar: (**a**) 5 mm. **Figure S3.** Variation in floral organ numbers, morphologies and structures of Oat-like rice. **Figure S4.** Sequence alignment of exon 1 of the *OsMADS1*^Olr^ or *OsMADS1* gene among Oat-like rice and representatively normal rice varieties. **Figure S5.** Protein sequence alignment of the MADS domain among OsMADS1^Olr^ or OsMADS1 and other MADS-Box family members in rice. **Figure S6.** Abnormal spikelet phenotypes of *OsMADS1*^Olr^***-***overexpressing plants in comparison with the wild-type plants. **Figure S7.** Abnormal spikelet phenotypes of *OsMADS1*-RNAi plants in comparison with the wild-type plants. **Figure S8.** Tissue-specific expression pattern of *OsMADS1* (**a** and **c**) and *OsTip3* (**b** and **d**), respectively. **a** and **b** Expression pattern of *OsMADS1* (**a**) and *OsTip3* (**b**) in various vegetative, reproductive tissues & organs, and seeds according to the microarray data released in the ePlant Rice database (http://bar.utoronto.ca/eplant_rice/). Color scale represents microarray signal level. P1: 0–3 cm inflorescence, floral transition and floral organ development; P2 and P3: 3–10 cm inflorescence, meiotic stage; P4: 10–15 cm inflorescence, young microspore stage; P5: 15–22 cm inflorescence, vacuolated pollen stage; P6: 22–30 cm inflorescence, mature pollen stage; Seed S1: 0–2 DAP, early globular embryo; Seed S2: 3–4 DAP, middle and late globular embryo; Seed S3: 5–10 DAP, embryo morphogenesis; Seed S4: 11–20 DAP, embryo maturation; Seed S5: 21–29 DAP, dormancy and desiccation tolerance; DAP: Days After Pollination; SAM: Shoot Apical Meristems. **c** and **d** Expression pattern of *OsMADS1* (**c**) and *OsTip3* (**d**) in various vegetative, reproductive tissues & organs, in addition to ovary, embryo and endosperm of seeds during different developmental stages according to the microarray data released in the Rice Expression Profile Database (RiceXPro) (https://ricexpro.dna.affrc.go.jp/category-select.php). The numbers 10,301 (**c**) and 02686 (**d**) indicate feature numbers of expression pattern of *OsMADS1* and *OsTip3* by using the corresponding probe, S-8726 (**c**) and S-2143 (**d**), respectively; DAF: Days After Fertilization. *OsMADS1* is expressed in inflorescences, spikelets, floral organs and seeds (**a** and **c**), and *OsTip3* is specifically expressed in seeds (**b** and **d**). **Figure S9.** Normal spikelet phenotypes of T_0_ p*OsTip3*::*OsMADS1*-RNAi plants (T3-Ri-6 (T_0_), T3-Ri-3 (T_0_), T3-Ri-4 (T_0_) and T3-Ri-7 (T_0_)) in comparison with wild-type (NIP). **a**-**o** Phenotypic analysis of spikelets and floral organs of NIP (**a**-**c**) and p*OsTip3*::*OsMADS1*-RNAi plants (**d**-**o**) by stereomicroscope. **a**, **d**, **g**, **j** and **m** A normal spikelet of NIP (**a**), T3-Ri-6 (T_0_) (**d**), T3-Ri-3 (T_0_) (**g**), T3-Ri-4 (T_0_) (**j**) and T3-Ri-7 (T_0_) (**m**). **b**, **e**, **h**, **k** and **n** A normal and dissected spikelet of NIP (**b**), T3-Ri-6 (T_0_) (**e**), T3-Ri-3 (T_0_) (**h**), T3-Ri-4 (T_0_) (**k**) and T3-Ri-7 (T_0_) (**n**) in which a lemma and a palea were ripped off, consisting of a pair of empty glumes, a pair of lodicules, six stamens and a pistil. **c**, **f**, **i**, **l** and **o** A normal pistil from a spikelet of NIP (**c**), T3-Ri-6 (T_0_) (**f**), T3-Ri-3 (T_0_) (**i**), T3-Ri-4 (T_0_) (**l**) and T3-Ri-7 (T_0_) (**o**) consists of an ovary and a pair of stigmas. eg, empty glume; le, lemma; pa, palea; lo, lodicule; st, stamen; pi, pistil; sti, stigma; ov, ovary. Bars: (**a**-**o**) 1 mm. **Figure S10.** Grain shape and relative expression of *OsMADS1* in 12 DAF grains of T_0_ p*OsTip3*::*OsMADS1*-RNAi plants (T3-Ri-6 (T_0_), T3-Ri-3 (T_0_), T3-Ri-4 (T_0_) and T3-Ri-7 (T_0_)) in comparison with wild-type (NIP). **a**-**d** Grain shape and phenotypes of T_0_ p*OsTip3*::*OsMADS1*-RNAi plants (T3-Ri-6 (T_0_) (**a**), T3-Ri-3 (T_0_) (**b**), T3-Ri-4 (T_0_) (**c**) and T3-Ri-7 (T_0_) (**d**)) in comparison with NIP. **e** Relative expression of *OsMADS1* in 12 DAF grains of wild-type and T_0_ p*OsTip3*::*OsMADS1*-RNAi plants (T3-Ri-6 (T_0_), T3-Ri-3 (T_0_), T3-Ri-4 (T_0_) and T3-Ri-7 (T_0_)). The value of *OsActin* mRNA was used as an internal control for data normalization, and the expression level of *OsMADS1* in (NIP) were set as 1.0. DAF, Days After Fertilization. Values are means ± SDs of three replicates. Student’s t-test: *p < 0.05; ***p < 0.001. Bars: (a-d) 5 mm. **Figure S11.** Grain shape and phenotypes of T_2_ p*OsTip3*::*OsMADS1*-RNAi lines (T3-Ri-5, T3-Ri-9 and T3-Ri-12) in comparison with grains of the negative control (NC) line, which shows the backside view of the grains shown in Fig. 11**a. Figure S12.** Schematic diagrams of the transgenic vectors. **Table S1.** Major agronomic traits of NIP and Oat-like rice. **Table S2.** Statistical analysis of variation in floral organ numbers of Oat-like rice. **Table S3.** Segregation analysis of F_2_ population derived from a cross combination between Oat-like rice and NIP. **Table S4.** PCR-based SSR and InDel molecular makers used and developed for fine mapping of the *OsMADS1*^Olr^ gene in rice. **Table S5.** Primers used for RT-PCR amplification and cDNA sequencing analysis of *OsMADS1* and *OsMADS*1^Olr^. **Table S6.** Primers used for qRT-PCR analysis. **Table S7.** Primers developed for vector construction.

**Additional file 2: Table S8.** Grain length of 141 representative rice varieties or accessions from 57 countries in the world in comparison with that of Oat-like rice.

## Data Availability

All data generated or analyzed during this study are included in this published article and its supplementary information files.

## Abbreviations

*AP1*: *APETALA1*
bHLH: Basic helix-loop-helix
*AG*: *AGAMOUS*
BSA: Bulked Segregate Analysis
*CAL*: *CAULIFLOWER*
ChIP-seq: Chromatin Immunoprecipitation Sequencing
DAF: Days after fertilization
DAP: Days after pollination
*DEP1*: *DENSE AND ERECT PANICLE 1*
*GS3*: *Grain length and weight 3*
*GS5*: *Grain size 5*
GSK2: Glycogen synthase kinase 2
GUS: β-glucuronidase
*GW2*: *Grain width and weight 2*
*GW5*: *qSW5*, *Grain width and weight*
*GW8*: *OsSPL16*, *Squamosa promoter-binding protein-like 16*
InDel: Insertion Deletion
*lhs1*: *Leafy hull sterile 1*
MCS: Multiple Cloning Sites
NIP: Nipponbare
*nsr*: *Naked seed rice*
*ohms1*: *Open hull and male sterile 1*
*OsBC1*: *Brassinosteroid upregulated 1-like 1* (*OsBUL1*) *complex 1*
*OsBU1*: *Brassinosteroid upregulated 1*
Plant TFDB: Plant Transcription Factor Database
qRT-PCR: Quantitative Real Time PCR
SEM: Scanning electron microscopy
SSR: Simple Sequence Repeat
SRF: Serum Response Factor
UTRs: Untranslated regions
