# Supplementary material for: Characterization of the ‘Oat-Like Rice’ Caused by a Novel Allele OsMADS1Olr Reveals Vital Importance of OsMADS1 in Regulating Grain Shape in Oryza sativa L
Source: Rice (N Y). 2020 Oct 15;13:73. doi: 10.1186/s12284-020-00428-x (PMC7561663; doi:10.1186/s12284-020-00428-x)
Supplement: Supplementary file 1 — Additional file 1: Figure S1. Morphologies of NIP and Oat-like rice at maturation stage. Figure S2. Grain shape (a) and grain length (b) of 23 representative rice varieties or accessions from 15 countries in the world in comparison with that of Oat-like rice. Bar: (a) 5 mm. Figure S3. Variation in floral organ numbers, morphologies and structures of Oat-like rice. Figure S4. Sequence alignment of exon 1 of the OsMADS1Olr or OsMADS1 gene among Oat-like rice and representatively normal rice varieties. Figure S5. Protein sequence alignment of the MADS domain among OsMADS1Olr or OsMADS1 and other MADS-Box family members in rice. Figure S6. Abnormal spikelet phenotypes of OsMADS1Olr-overexpressing plants in comparison with the wild-type plants. Figure S7. Abnormal spikelet phenotypes of OsMADS1-RNAi plants in comparison with the wild-type plants. Figure S8. Tissue-specific expression pattern of OsMADS1 (a and c) and OsTip3 (b and d), respectively. a and b Expression pattern of OsMADS1 (a) and OsTip3 (b) in various vegetative, reproductive tissues & organs, and seeds according to the microarray data released in the ePlant Rice database (http://bar.utoronto.ca/eplant_rice/). Color scale represents microarray signal level. P1: 0–3 cm inflorescence, floral transition and floral organ development; P2 and P3: 3–10 cm inflorescence, meiotic stage; P4: 10–15 cm inflorescence, young microspore stage; P5: 15–22 cm inflorescence, vacuolated pollen stage; P6: 22–30 cm inflorescence, mature pollen stage; Seed S1: 0–2 DAP, early globular embryo; Seed S2: 3–4 DAP, middle and late globular embryo; Seed S3: 5–10 DAP, embryo morphogenesis; Seed S4: 11–20 DAP, embryo maturation; Seed S5: 21–29 DAP, dormancy and desiccation tolerance; DAP: Days After Pollination; SAM: Shoot Apical Meristems. c and d Expression pattern of OsMADS1 (c) and OsTip3 (d) in various vegetative, reproductive tissues & organs, in addition to ovary, embryo and endosperm of seeds during different developmental stages a [file 12284_2020_428_MOESM1_ESM.doc]

**Additional file 1:**

**
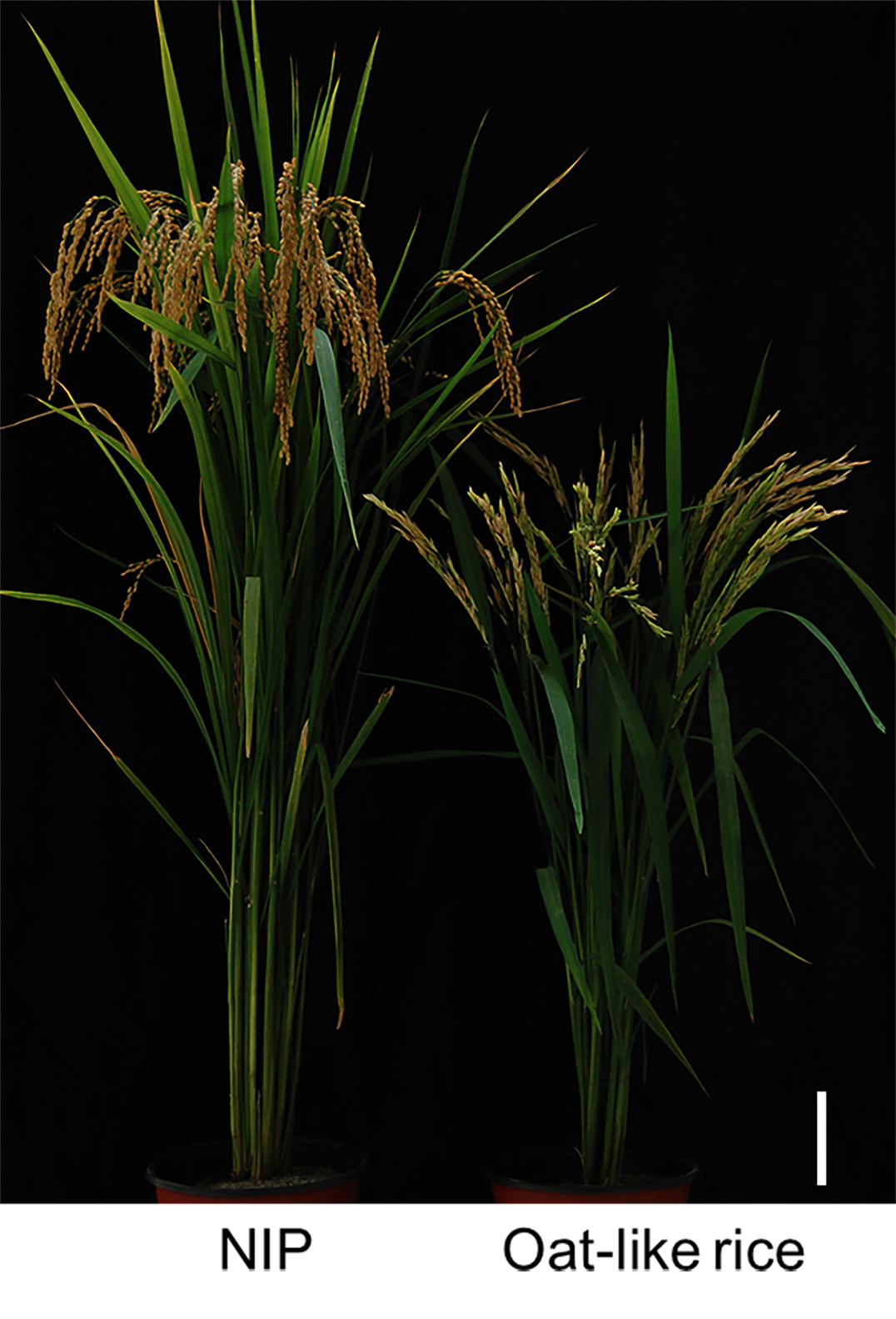
**

**Figure S1.** Morphologies of NIP (Nipponbare) and Oat-like rice at maturation stage. NIP (left) and Oat-like rice (right) plants. Bar: 10 cm.


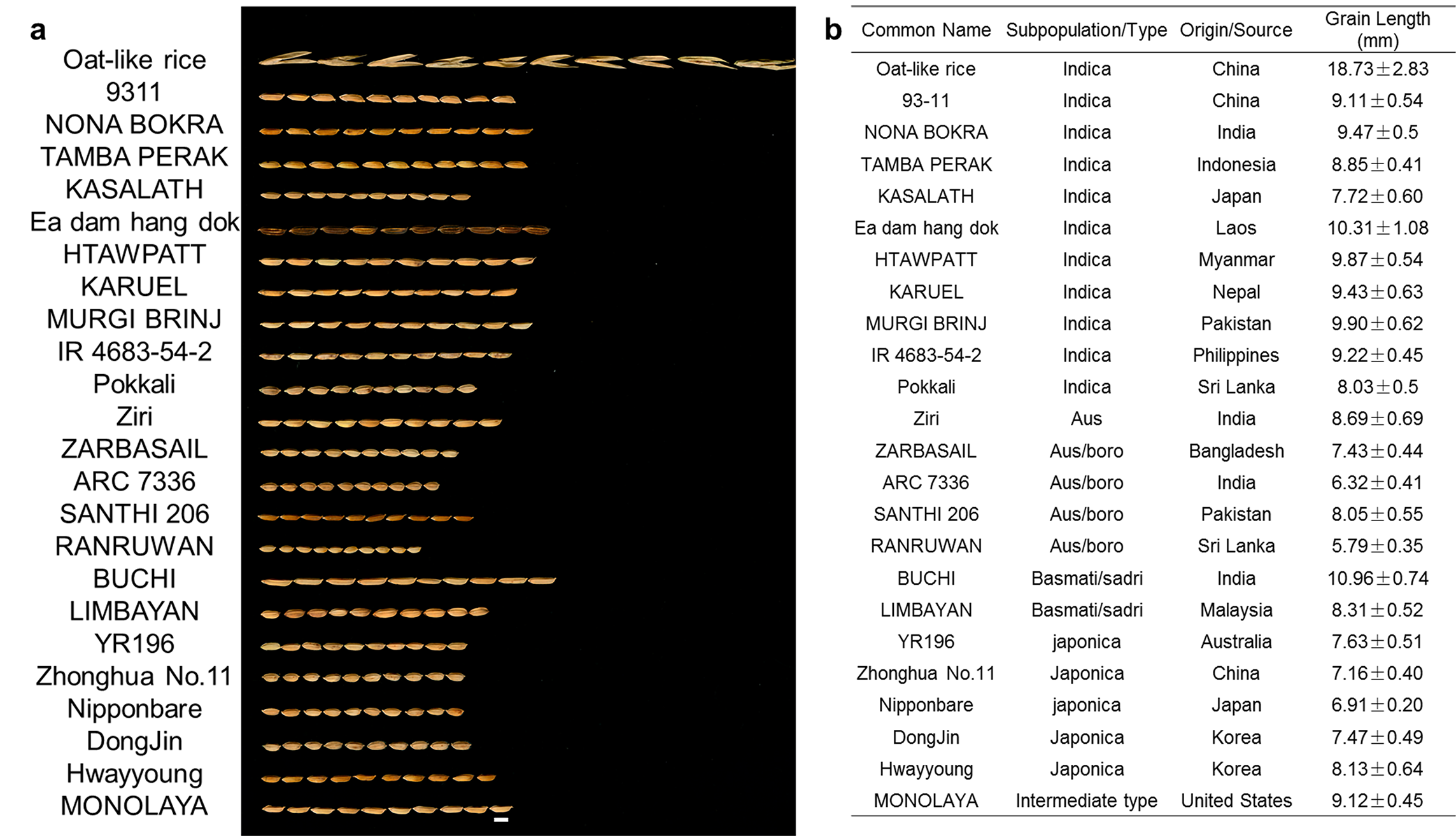


**Figure S2.** Grain shape (**a**) and grain length (**b**) of 23 representative rice varieties or accessions from 15 countries in the world in comparison with that of Oat-like rice. Bar: (**a**) 5 mm.


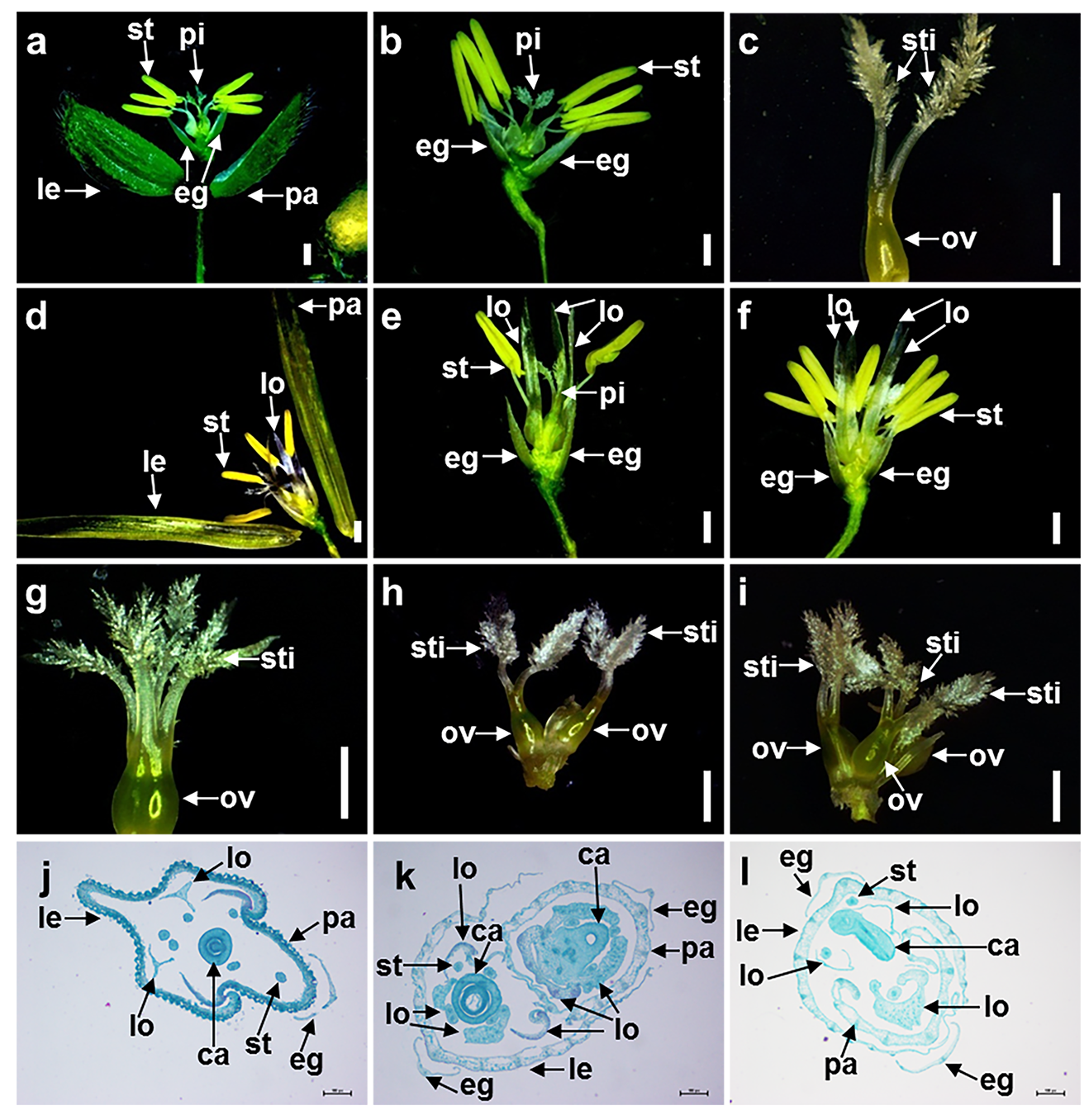


**Figure S3.** Variation in floral organ numbers, morphologies and structures of Oat-like rice**. a-i** Phenotype analysis of spikelets and floral organs of NIP (**a**-**c**) and Oat-like rice (**d**-**i**) by stereomicroscope. **a** A normal and dissected spikelet of NIP consists of a pair of empty glumes, a lemma, a palea, a pair of lodicules, six stamens and a pistil. **b** A dissected spikelet of NIP in which a lemma and a palea were ripped off. **c** A normal pistil from a spikelet of NIP consists of an ovary and a pair of stigmas. **d** A dissected abnormal spikelet of Oat-like rice consists of a pair of empty glumes, an extremely elongated and leafy lemma and palea respectively, a pair of extremely elongated lodicules, four stamens and a pistil. **e** A dissected spikelet of Oat-like rice in which a lemma and a palea were ripped off, consisting of three extremely elongated lodicules, two stamens and a pistil. **f** A dissected spikelet of Oat-like rice in which a lemma and a palea were ripped off, consisting of four extremely elongated lodicules, seven stamens and two pistils. **g** A pistil from a spikelet of Oat-like rice consists of an ovary and four stigmas. **h** A pistil from a spikelet of Oat-like rice shows two intact ovaries and four stigmas. **h** and **i** Conjugated twin pistils (**h**) and conjugated triplet pistils (**i**) in spikelets of Oat-like rice. **j-l** Transverse sections of spikelets of NIP (**j**) and Oat-like rice (**k** and **l** ) by paraffin sections. **j** A transverse-sectioned spikelet of NIP. The transverse section shows an empty glume, a lemma, a palea, a pair of lodicules, six stamens and a carpel. **k** A transverse-sectioned spikelet of Oat-like rice. The transverse section shows a pair of empty glume, an abnormal lemma and palea respectively, six abnormally leafy lemma- and palea- like lodicules, four stamens and two carpels. **l** A transverse-sectioned spikelet of Oat-like rice. The transverse section shows a pair of empty glume, an abnormal lemma and palea respectively, three abnormally leafy lemma- and palea-like lodicules, three stamens and an abnormal carpel. eg, empty glume; le, lemma; pa, palea; lo, lodicule; st, stamen; pi, pistil; ca, carpel; sti, stigma; ov, ovary. Bars: (**a**-**i**) 1 mm; (**j**-**l**) 100 μm.


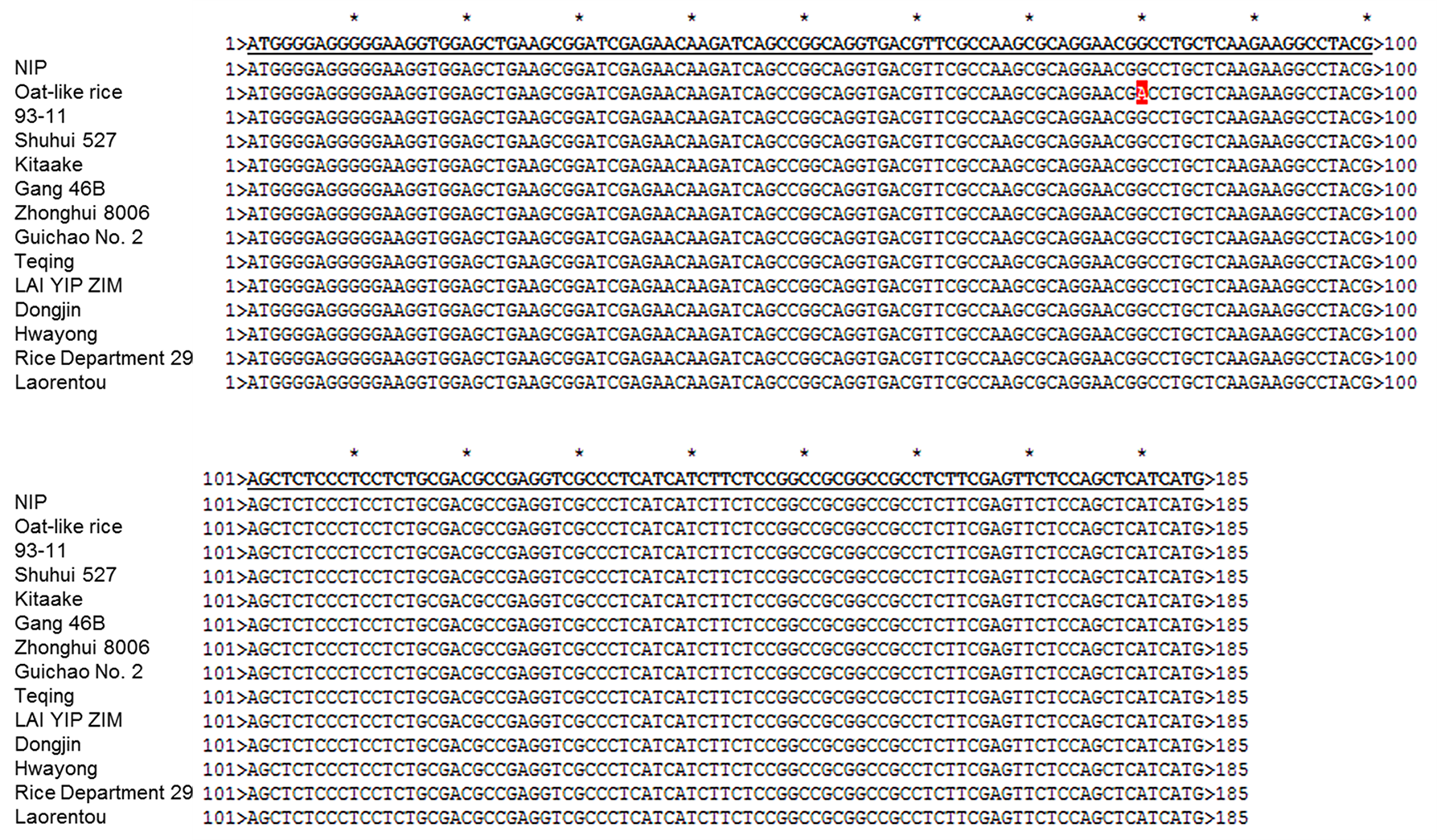


**Figure S4.** Sequence alignment of exon 1 of the *OsMADS1*^Olr^ or *OsMADS1* gene among Oat-like rice and representatively normal rice varieties**.** The substitution base in exon 1 of the *OsMADS1*^Olr^ allele in Oat-like rice is highlighted with the red color.


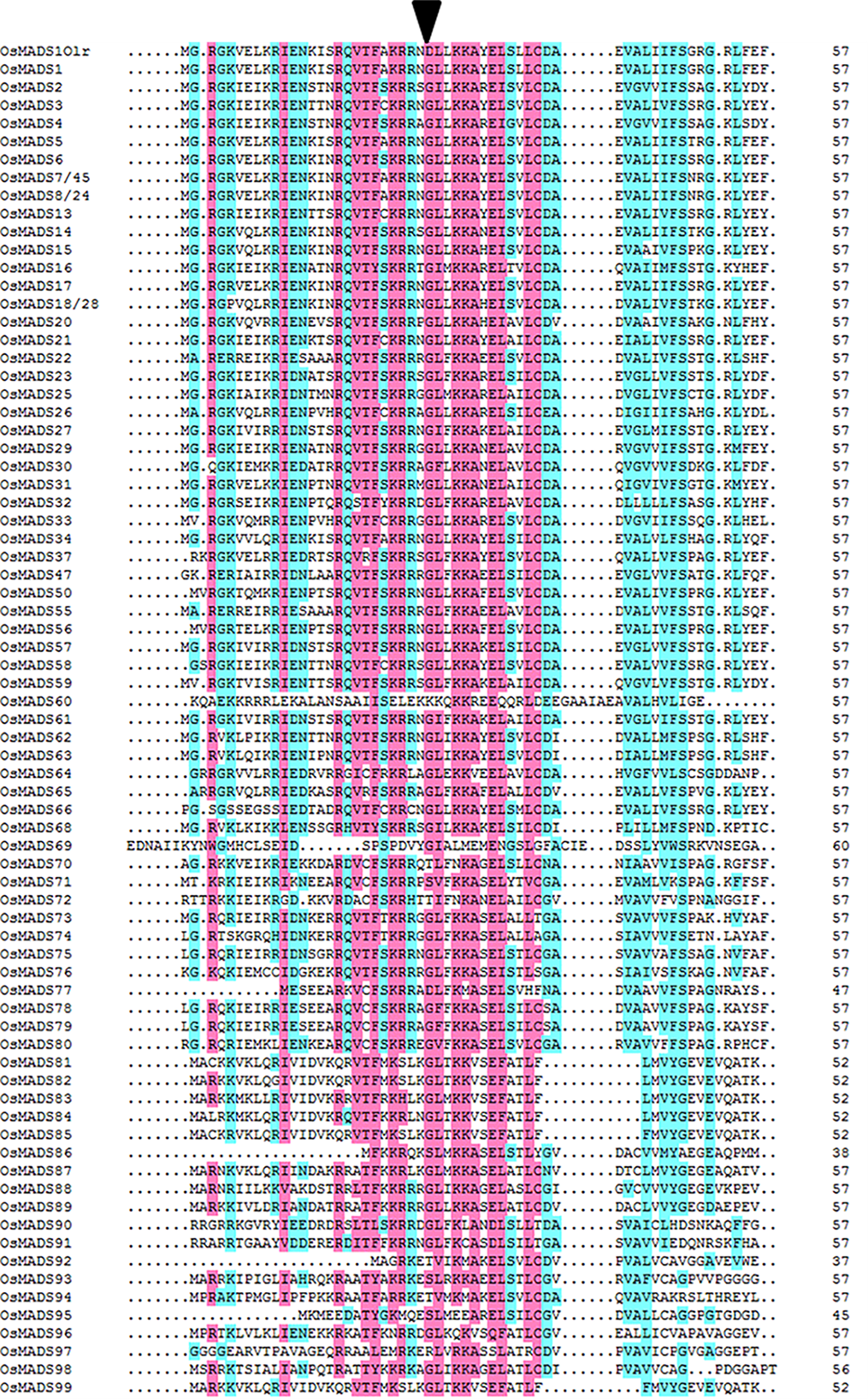


**Figure S5.** Protein sequence alignment of the MADS domain among OsMADS1^Olr^ or OsMADS1 and other MADS-Box family members in rice. The substitution site in the 27th amino acid of the MADS domain of OsMADS1^Olr^ protein is indicated by the black arrowhead.


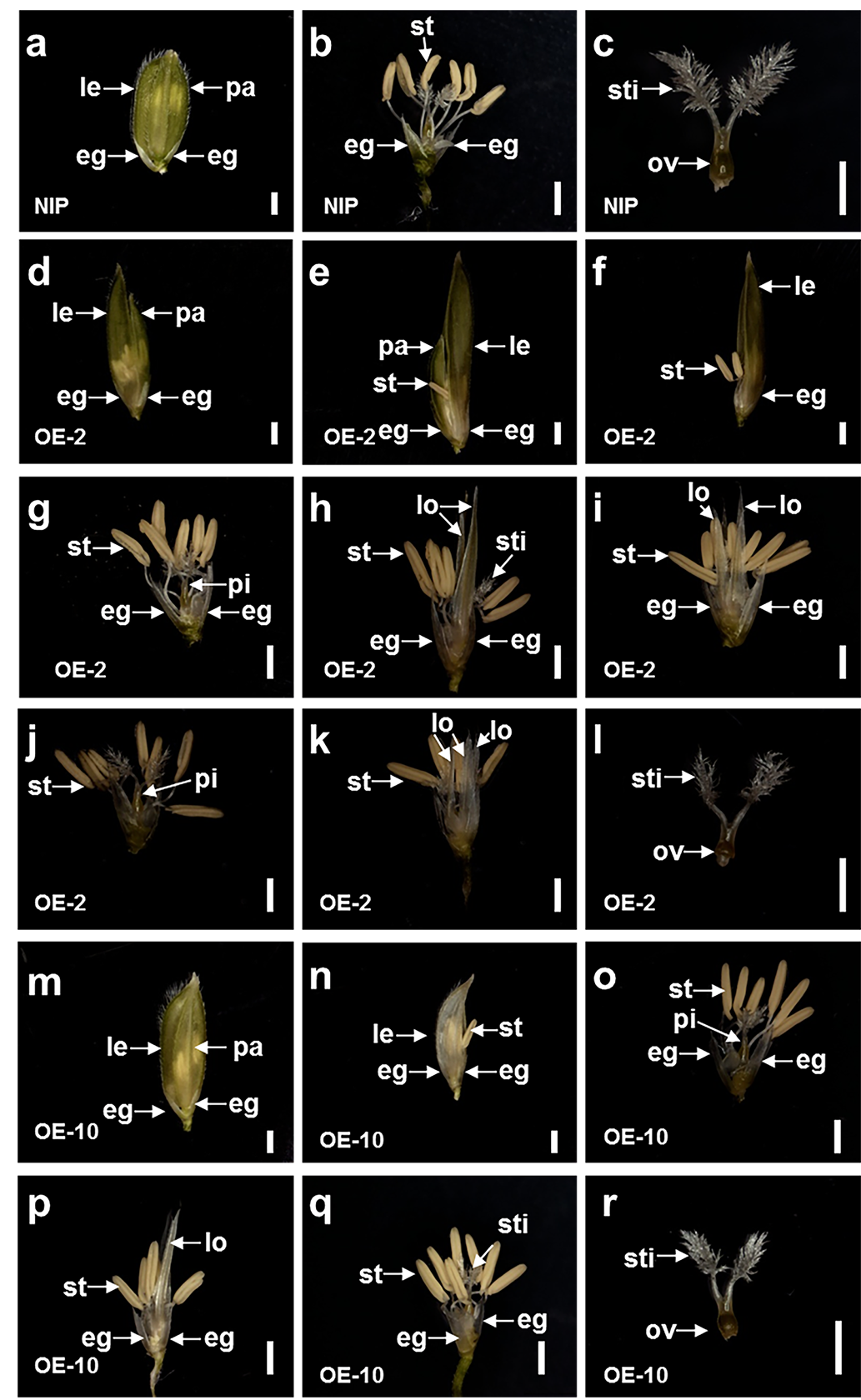


**Figure S6.** Abnormal spikelet phenotypes of *OsMADS1*^Olr^***-***overexpressing plants in comparison with wild-type plants. **a-c** Spikelet and floral organs of the wild-type plant (NIP). **a** A normal spikelet of NIP. **b** A normal and dissected spikelet of NIP in which a lemma and a palea were ripped off, consisting of a pair of empty glumes, a pair of lodicules, six stamens and a pistil. **c** A pistil from a spikelet of NIP consists of an ovary and a pair of stigmas. **d-l** Spikelets and floral organs of a *OsMADS1*^Olr^***-***overexpressing plant with severe phenotypes, OE-2. **d** and **e** Abnormal spikelets of OE-2 showing open hull. **f** An abnormal spikelet of OE-2, which has no palea. **g** A dissected spikelet of OE-2 in which a lemma and a palea were ripped off, exhibiting six stamens and a pistil. **h** A dissected abnormal spikelet of OE-2 in which a lemma and a palea were ripped off, exhibiting five stamens and two aberrant extremely elongated lodicules. **i** A dissected abnormal spikelet of OE-2 in which a lemma and a palea were ripped off, exhibiting eight stamens and three aberrant extremely elongated lodicules. **j** A dissected abnormal spikelet of OE-2 in which a lemma and a palea were ripped off, exhibiting seven stamens and a pistil. **k** A dissected abnormal spikelet of OE-2 in which a lemma and a palea were ripped off, exhibiting four stamens and three aberrant extremely elongated lodicules. **l** A pistil from a spikelet of OE-2 consists of an ovary and a pair of stigmas. **m-r** Spikelets and floral organs of the *OsMADS1*^Olr^***-***overexpressing plant with weak phenotypes, OE-10. **m** Compared with wild-type spikelet, a more slender spikelet of OE-10 shows closed hull. **n** An abnormal spikelet of OE-10, which has no palea. **o** A dissected spikelet of OE-10 in which a lemma and a palea were ripped off, exhibiting six stamens and a pistil. **p** A dissected abnormal spikelet of OE-10 in which a lemma and a palea were ripped off, exhibiting five stamens and two aberrant extremely elongated lodicules. **q** A dissected abnormal spikelet of OE-10 in which a lemma and a palea were ripped off, exhibiting seven stamens and a pistil. **r** A pistil from a spikelet of OE-10 consists of an ovary and a pair of stigmas. eg, empty glume; le, lemma; pa, palea; lo, lodicule; st, stamen; pi, pistil; sti, stigma; ov, ovary. Bars: (**a**-**r**) 1 mm.


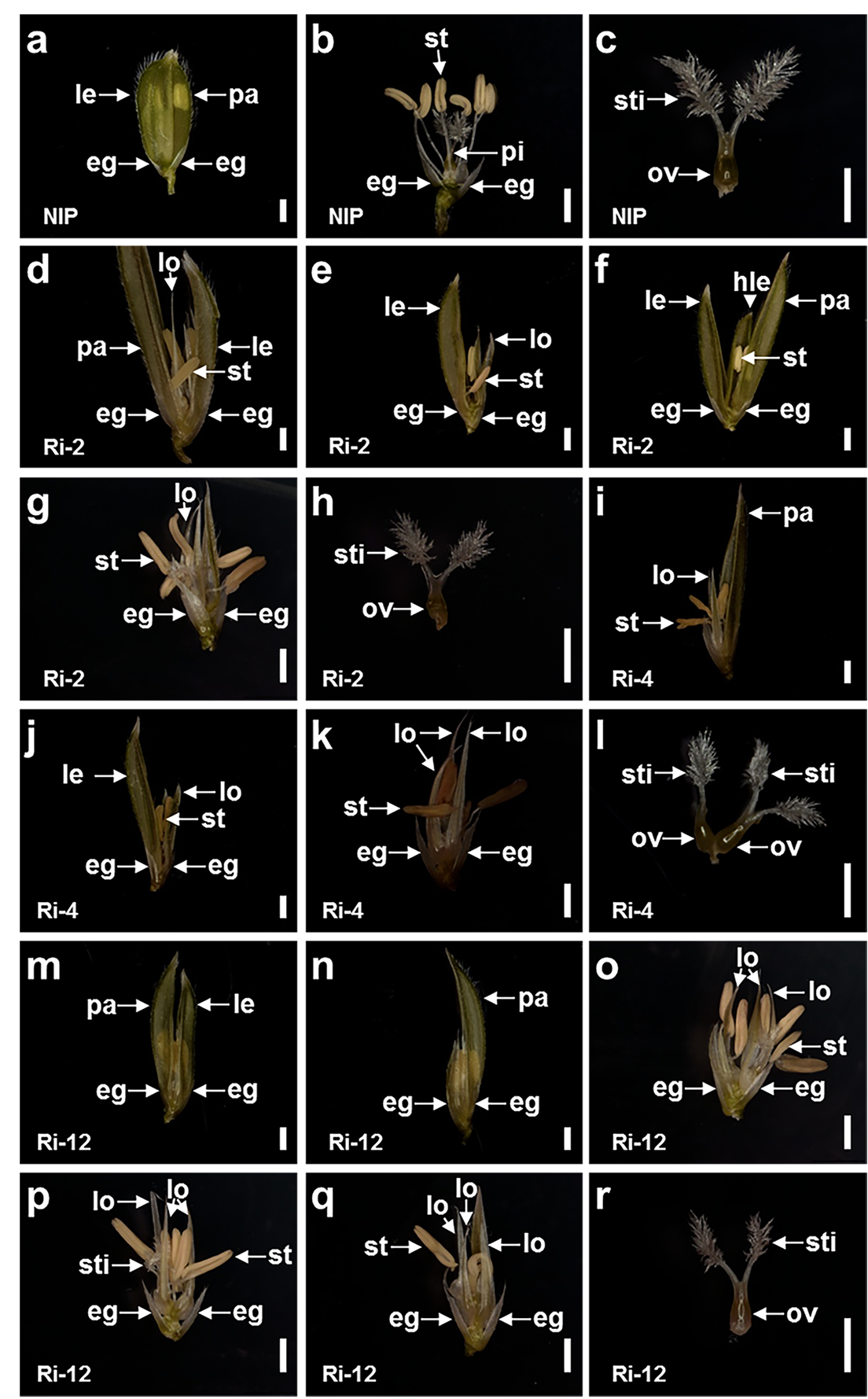


**Figure S7.** Abnormal spikelet phenotypes of *OsMADS1*-RNAi plants in comparison with the wild-type plants. **a-c** Spikelet and floral organs of the wild-type plant (NIP). **a** A normal spikelet of NIP. **b** A normal and dissected spikelet of NIP in which a lemma and a palea were ripped off, consisting of a pair of empty glumes, a pair of lodicules, six stamens and a pistil. **c** A pistil from a spikelet of NIP consists of an ovary and a pair of stigmas. **d-h** Spikelets and floral organs of the *OsMADS1*-RNAi plant with severe phenotypes, Ri-2. **d-f** Abnormal spikelets of Ri-2 showing open hull. A hyperplastic lemma is shown by an arrowhead in **f**. **g** A dissected spikelet of Ri-2 in which a lemma and a palea were ripped off, exhibiting five stamens and three aberrant extremely elongated lodicules. **h** A pistil from a spikelet of Ri-2 consists of an ovary and a pair of stigmas. **i-l** Spikelets and floral organs of the *OsMADS1*-RNAi plant with severe phenotypes, Ri-4. **i** and **j** Abnormal spikelet of Ri-2 showing open hull. **k** A dissected spikelet of Ri-4 in which a lemma and a palea were ripped off, exhibiting two stamens and three aberrant extremely elongated lodicules. **l** Aberrant conjugated twin pistils from a spikelet of Ri-4. **m-r** Spikelets and floral organs of the *OsMADS1*-RNAi plant with severe phenotypes, Ri-12. **m** and **n** Abnormal spikelets of Ri-12 showing open hull. **o** A dissected spikelet of Ri-12 in which a lemma and a palea were ripped off, exhibiting six stamens and three aberrant extremely elongated lodicules. **p** A dissected spikelet of Ri-12 in which a lemma and a palea were ripped off, exhibiting five stamens and three aberrant extremely elongated lodicules. **q** A dissected spikelet of Ri-12 in which a lemma and a palea were ripped off, exhibiting two stamens and three aberrant extremely elongated lodicules. **r** A pistil from a spikelet of Ri-12 consists of an ovary and a pair of stigmas. eg, empty glume; le, lemma; hle, hyperplastic lemma; pa, palea; lo, lodicule; st, stamen; pi, pistil; sti, stigma; ov, ovary. Bars: (**a**-**r**) 1 mm.


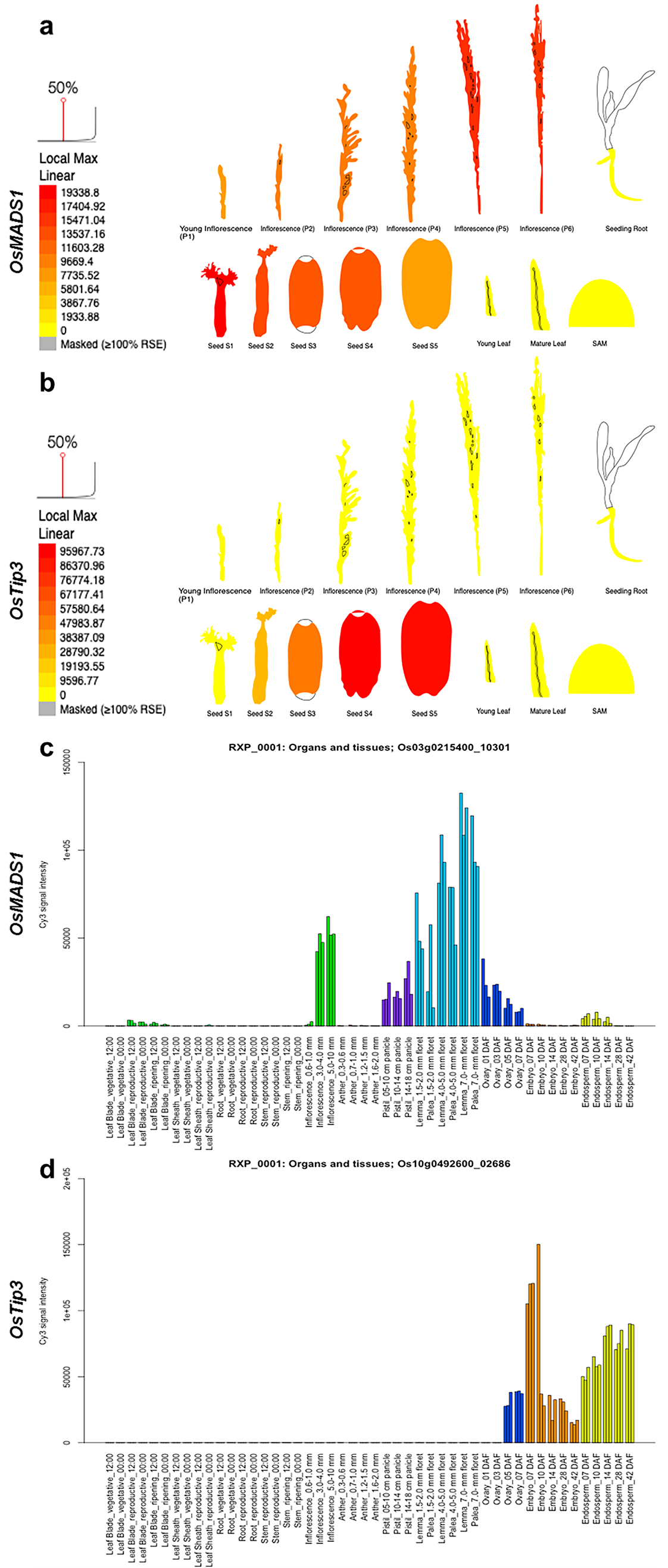


**Figure S8**. Tissue-specific expression pattern of *OsMADS1* (**a** and **c**) and *OsTip3* (**b** and **d**), respectively. **a** and **b** Expression pattern of *OsMADS1* (**a**) and *OsTip3* (**b**) in various vegetative, reproductive tissues & organs, and seeds according to the microarray data released in the ePlant Rice database (http://bar.utoronto.ca/eplant_rice/). Color scale represents microarray signal level. P1: 0-3 cm inflorescence, floral transition and floral organ development; P2 and P3: 3-10 cm inflorescence, meiotic stage; P4: 10-15 cm inflorescence, young microspore stage; P5: 15-22 cm inflorescence, vacuolated pollen stage; P6: 22-30 cm inflorescence, mature pollen stage; Seed S1: 0-2 DAP, early globular embryo; Seed S2: 3-4 DAP, middle and late globular embryo; Seed S3: 5-10 DAP, embryo morphogenesis; Seed S4: 11-20 DAP, embryo maturation; Seed S5: 21-29 DAP, dormancy and desiccation tolerance; DAP: Days After Pollination; SAM: Shoot Apical Meristems. **c** and **d** Expression pattern of *OsMADS1* (**c**) and *OsTip3* (**d**) in various vegetative, reproductive tissues & organs, in addition to ovary, embryo and endosperm of seeds during different developmental stages according to the microarray data released in the Rice Expression Profile Database (RiceXPro) (https://ricexpro.dna.affrc.go.jp/category-select.php). The numbers 10301 (**c**) and 02686 (**d**) indicate feature numbers of expression pattern of *OsMADS1* and *OsTip3* by using the corresponding probe, S-8726 (**c**) and S-2143 (**d**), respectively; DAF: Days After Fertilization. *OsMADS1* is expressed in inflorescences, spikelets, floral organs and seeds (**a** and **c**), and *OsTip3* is specifically expressed in seeds (**b** and **d**).


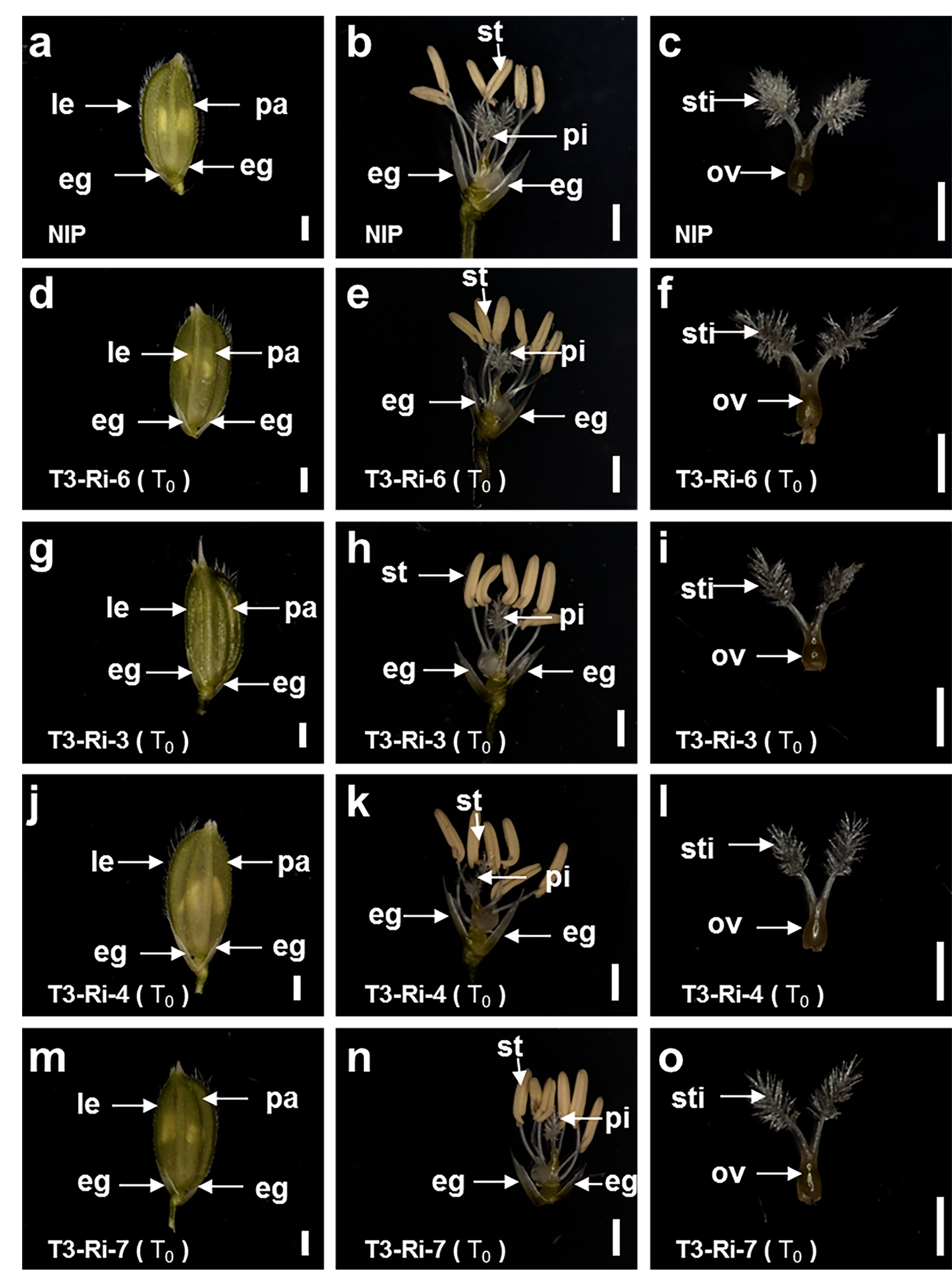


**Figure S9**. Normal spikelet phenotypes of T_0_ p*OsTip3*::*OsMADS1*-RNAi plants (T3-Ri-6 (T_0_), T3-Ri-3 (T_0_), T3-Ri-4 (T_0_) and T3-Ri-7 (T_0_)) in comparison with wild-type (NIP). **a**-**o** Phenotypic analysis of spikelets and floral organs of NIP (**a**-**c**) and p*OsTip3*::*OsMADS1*-RNAi plants (**d**-**o**) by stereomicroscope. **a**, **d**, **g**, **j** and m A normal spikelet of NIP (**a**), T3-Ri-6 (T_0_) (**d**), T3-Ri-3 (T_0_) (**g**), T3-Ri-4 (T_0_) (**j**) and T3-Ri-7 (T_0_) (**m**). **b**, **e**, **h**, **k** and **n** A normal and dissected spikelet of NIP (**b**), T3-Ri-6 (T_0_) (**e**), T3-Ri-3 (T_0_) (**h**), T3-Ri-4 (T_0_) (**k**) and T3-Ri-7 (T_0_) (**n**) in which a lemma and a palea were ripped off, consisting of a pair of empty glumes, a pair of lodicules, six stamens and a pistil. **c**, **f**, **i,** l and **o** A normal pistil from a spikelet of NIP (**c**), T3-Ri-6 (T_0_) (**f**), T3-Ri-3 (T_0_) (**i**), T3-Ri-4 (T_0_) (**l**) and T3-Ri-7 (T_0_) (**o**) consists of an ovary and a pair of stigmas. eg, empty glume; le, lemma; pa, palea; lo, lodicule; st, stamen; pi, pistil; sti, stigma; ov, ovary. Bars: (**a**-**o**) 1 mm.


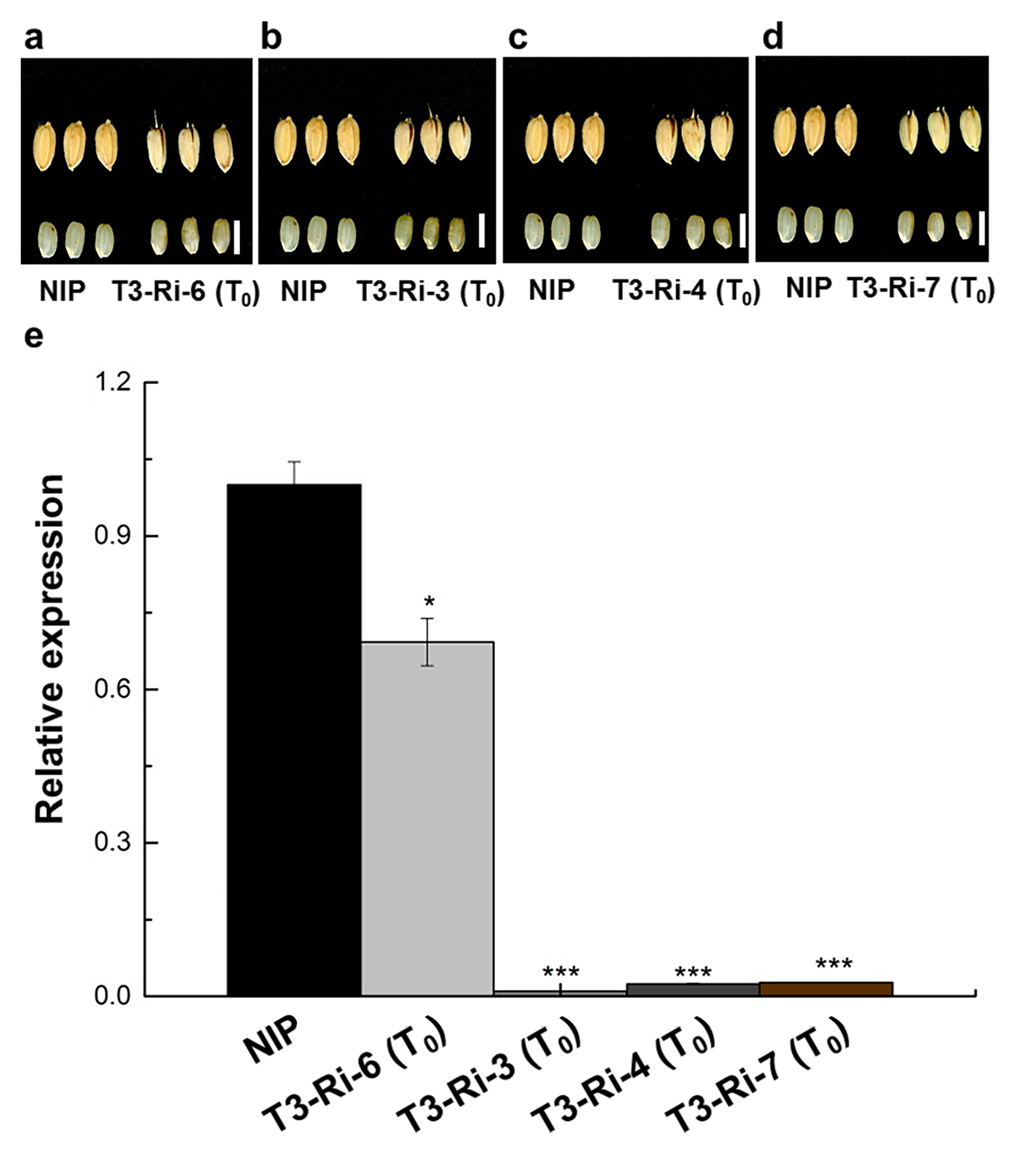


**Figure S10**. Grain shape and relative expression of *OsMADS1* in 12 DAF grains of T_0_ p*OsTip3*::*OsMADS1*-RNAi plants (T3-Ri-6 (T_0_), T3-Ri-3 (T_0_), T3-Ri-4 (T_0_) and T3-Ri-7 (T_0_)) in comparison with wild-type (NIP). **a**-**d** Grain shape and phenotypes of T_0_ p*OsTip3*::*OsMADS1*-RNAi plants (T3-Ri-6 (T_0_) (**a**), T3-Ri-3 (T_0_) (**b**), T3-Ri-4 (T_0_) (**c**) and T3-Ri-7 (T_0_) (**d**)) in comparison with NIP. **e** Relative expression of *OsMADS1* in 12 DAF grains of wild-type and T_0_ p*OsTip3*::*OsMADS1*-RNAi plants (T3-Ri-6 (T_0_), T3-Ri-3 (T_0_), T3-Ri-4 (T_0_) and T3-Ri-7 (T_0_)). The value of *OsActin* mRNA was used as an internal control for data normalization, and the expression level of *OsMADS1* in (NIP) were set as 1.0. DAF, Days After Flowering. Values are means ± SDs of three replicates. Student’s t-test: *p<0.05; ***p<0.001.


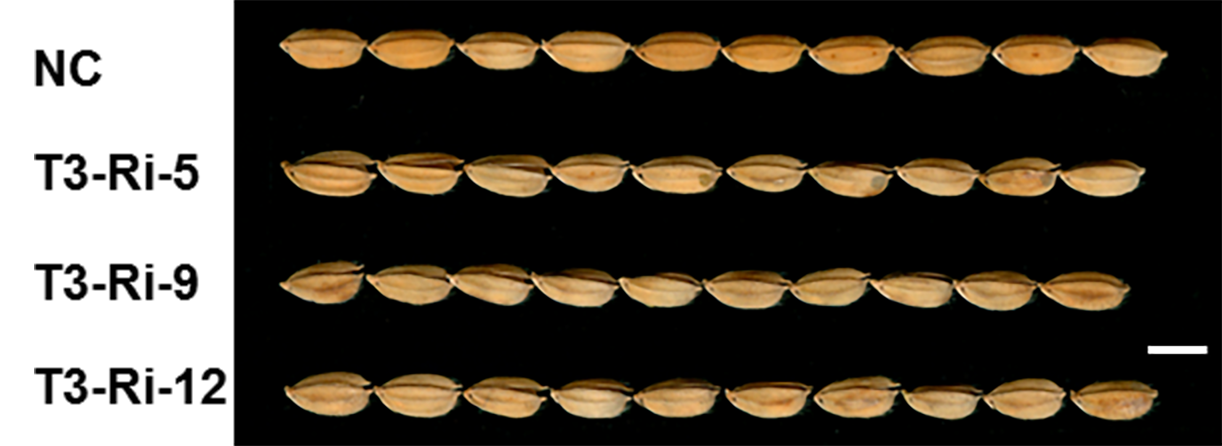


**Figure S11**. Grain shape and phenotypes of T_2_ p*OsTip3*::*OsMADS1*-RNAi lines (T3-Ri-5, T3-Ri-9 and T3-Ri-12) in comparison with grains of the negative control (NC) line, which shows the backside view of the grains shown in Fig. 11a


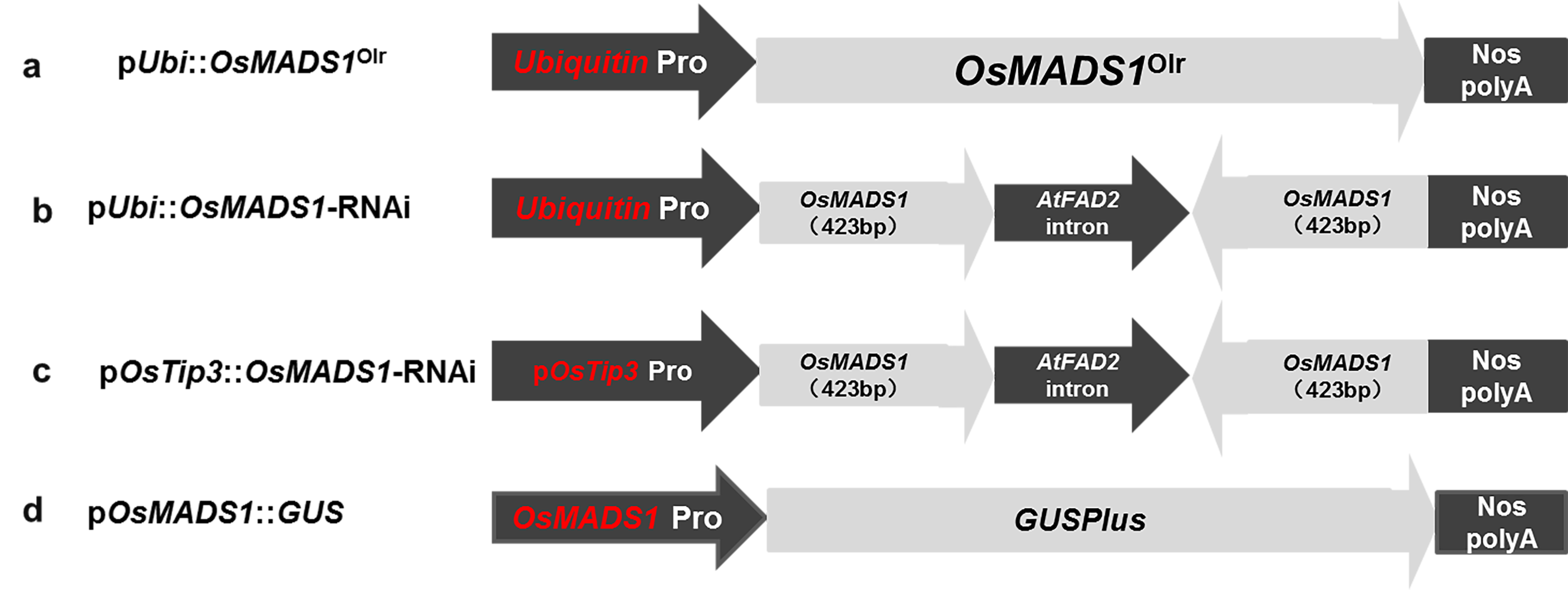


**Figure S12.** Schematic diagrams of the transgenic vectors. **a** p*Ubi*::*OsMADS1*^Olr^, *OsMADS1*^Olr^***-***overexpressing vector. **b** p*Ubi*::*OsMADS1*-RNAi, *OsMADS1*-RNAi vector. **c** p*OsTip3*::*OsMADS1*-RNAi, Seed-specifically RNAi vector for *OsMADS1* driven by the Seed-specific promoter, p*OsTip3*. **d** p*OsMADS1*::*GUS*, *GUS* expressing vector driven by *OsMADS1* promoter. *AtFAD2* represents *fatty acid desaturase* gene in *Arabidopsis* (**b** and **c**)*.*

**Table S1.** Major agronomic traits of NIP and Oat-like rice

| Major agronomic traits | NIP | Oat-like rice | n |
| --- | --- | --- | --- |
| Plant height (cm) | 98.49±4.88 | 80.63±6.74*** | 60 |
| Number of tillers per plant | 18.32±6.82 | 16.66±6.71 | 60 |
| Panicle length (cm) | 20.47±1.26 | 20.46±1.40 | 24 |
| Total grain number per panicle | 130.04±12.22 | 63.96±11.05*** | 24 |

Data presented are mean values ± SDs. The ‘n’ represents number of samples used for the statistical analysis. Student’s *t*-test: ^***^p < 0.001.

**Table S2.** Statistical analysis of variation in floral organ numbers of Oat-like rice

| Floral organs | Number | Percentage (%) |
| --- | --- | --- |
| Empty glume | 2 | 94.44 |
|  | 3 | 5.56 |
| Lemma and palea | 2 | 97.22 |
|  | 3 | 2.78 |
| Lodicule | 2 | 8.33 |
|  | 3 | 38.89 |
|  | 4 | 44.44 |
|  | 5 | 5.56 |
|  | 6 | 2.78 |
| Stamen | 2 | 19.44 |
|  | 3 | 36.11 |
|  | 4 | 30.56 |
|  | 5 | 5.56 |
|  | 6 | 5.56 |
|  | 7 | 2.78 |
| Pistil | 1 | 22.22 |
|  | 2 | 33.33 |
|  | 3 | 38.89 |
|  | 4 | 5.56 |
| Stigma | 1 | 4.88 |
|  | 2 | 85.37 |
|  | 3 | 7.32 |
|  | 4 | 2.44 |
| Ovary | 1 | 100.00 |

Data presented are values obtained from 36 mature spikelets. The 36 mature spikelets are obtained from 12 panicles (each three mature florets per panicle). Number of floral organs in the table means number of empty glume, lemma and palea, lodicule, stamen and pistil in a spikelet respectively, and number of stigma and ovary in a pistil respectively.

**Table S3.** Segregation analysis of F_2_ population derived from a cross combination between Oat-like rice and NIP

| Cross combination | Phenotype of F_1_ | F_2_ | | |  |  |
| --- | --- | --- | --- | --- | --- | --- |
|  |  | No. of plants showing normal phenotype | No. of plants showing Oat-like rice phenotype | Total plants | X^2^ _(3:1)_ | *P*-value |
| Oat-like rice / NIP | Normal phenotype | 2180 | 1450 | 3630 | 432.56 | ‹0.001 |

*P* value was determined with a Student’s *t*-test analysis.

**Table S4.** PCR-based SSR and InDel molecular makers used and developed for fine mapping of the *OsMADS1*^Olr^ gene in rice

| Markers | Forward primers (5′–3′) | Reverse primers (5′–3′) | Product size in NIP (bp) | Product size in 93-11 (bp) |
| --- | --- | --- | --- | --- |
| InDel 3-2 | TACTTTAATTTTGCAGCTC | TTTTACCCCACTCCATCT | 199 | 191 |
| InDel 3-4 | GCTTACCACACCTCTCCTCCT | TCCATATGCTTCCTTCTTCCA | 174 | 173 |
| InDel 3-7 | CTGCACCGGAGAAATTTGAT | CGCATGCAGATGAATAGGTG | 215 | 202 |
| InDel 3-11 | GGAATCCCTCCCTTCTTGTC | GGTCGGTAAAGACGGTGAAA | 140 | 129 |
| InDel 3-12 | CCAGGGATCTTCTCATCCAA | CCTGGCTAGCATACCACACA | 170 | 189 |
| InDel 3-13 | GCCATTGATCTTCTGCAGGT | TTTGTTGTCAATGCCCTGTT | 153 | 138 |
| InDel 3-14 | TATAGCGGACTGGCCAAACT | CCACCCATGTCATCTTCCAT | 195 | 207 |
| InDel 3-16 | CGACGCTGTTGATCCTGTTA | GAAATTAAGCAGCGGAAGCA | 171 | 155 |
| InDel 3-21 | GCGAGATGGGCAGCTACTAC | ACACAATGTCCAGCTTGCAG | 138 | 15` |
| RM3864 | AGTCAACCTTGGGGGTAAGG | AGATACTGCCCGTGTCATCC | 174 | 152 |
| RM7576 | CTGCCCTGCCTTTTGTACAC | GCGAGCATTCTTTCTTCCAC | 205 | 221 |
| FMM-3 | AGGACGATGGAGGCAGTTGT | AATGTCTTTCAACACCGCACG | 102 | 93 |
| FMM-17 | AGTATGTACCCCCTATAAGACCCAG | AACTTGAACACATGACATATACTTTGC | 99 | 150 |
| FMM-23 | GCCTCTTCGAGTTCTCCAGCTC | CTGCTGCGCCGGAACAAG | 89 | 91 |
| FMM-30 | AAGGCGAGGGAAAAAAAAAACA | CTTGTATGGCAGGAGAGTGGTGA | 233 | 0 |

**Table S5.** Primers used for RT-PCR amplification and cDNA sequencing analysis of *OsMADS1* and *OsMADS1*Olr

| Primer pair | Forward primer (5′–3′) | Reverse primer (5′–3′) | Product size in NIP (bp) |
| --- | --- | --- | --- |
| *OsMADS1*-RT | TTCGCCAAGCGCAGGGTCG | GGAGCTGCTGCATCCTGTGAGTT | 188 |

**Table S6.** Primers used for qRT-PCR analysis

| Primers | Forward primers (5′–3′) | Reverse primers (5′–3′) | Product size in NIP (bp) |
| --- | --- | --- | --- |
| *OsActin* | AGGAAGGCTGGAAGAGGACC | CGGGAAATTGTGAGGGACAT | 181 |
| *OsMADS1* | CTACATGGACCATCTGAGCAATGA | AAGAGAGCACGCACGTACTTAG | 222 |
| *GS3* | GAACTCCTGATCCATTCATAACGATT | CAAACAGCGAAACTTCTTCAAGAA | 161 |
| *GS5* | CATTCCATGCAAATGCCAGTGGAC | CAGCCCTGCTTTGATGAGCTTG | 230 |
| *GW2* | CAGCAGCGCATTCCCAGTTTTC | GTGGTCAGCCGAGCACTCTC | 159 |
| *GW8* | AGGAGTTTGATGAGGCCAAG | GCGTGTAGTATGGGCTCTCC | 407 |
| *GW5* | TGGGATATGGAATGGAATGGGTTGG | GATAGGGGTGGGGATGGGATGAATG | 354 |
| *RGB1* | GCTGCCTTGGTCTTTCTTCT | GGCCCAAATCTTCAAATTCT | 273 |
| *DEP1* | ATGCCCACGGTGTCGTAAC | TCGAACTTAATCAAAGGCCTAA | 115 |
| *GGC2* | GTGCAACTGCTTGTTATGCC | GCTCGGTCTACAGCACGAT | 117 |
| *OsBU1* | CTCATCTCTTCTCATCTGTTCTTC | ATCAGTAGTACACCGAGATGAGTA | 207 |
| *OsBC1* | AGTAGGGGAGCCAAGGCAAG | CAGACAAGGGGATGGACTCG | 401 |

**Table S7.** Primers developed for vector construction

| Primers | Final vectors | Inserted vectors  (Original vectors) | Forward primers (5′–3′) and reverse primers (5′–3′) |
| --- | --- | --- | --- |
| OE-1 | p*Ubi*::*OsMADS1*^Olr^ | pC*Ubi*1390 | F: CGCGGATCCATCAGGTAGCCAAACCACACCAC  R: GGACTAGTTGCCAATTAACTTGTTACCACATCC |
| Ri-1 | p*Ubi*::*OsMADS1*-RNAi | pLHRNAi | F: TTCTGCACTAGGTACCAGGCCTGAACAAATCAGGTCAAGAAAG  R: CTGACGTAGGGGCGATAGAGCTCTGTTTGCATTGGCTTCT |
| Ri-2 |  |  | F: CGGGGATCCGTCGACTACAACAAATCAGGTCAAGAAAG  R: AGGTGGAAGACGCGTTACTGTTTGCATTGGCTTCT |
| T3-Ri-1 | p*OsTip3*:*OsMADS1*-RNAi | pLHRNAi | F: TGGGCCCGGCGCGCCAAGCTTCTCTGCACAGAATTTGCCGG  R: ATAGAGCTCAGGCCTGGTACCACCGATCACTCACTCTCACTACTCAC |
| T3-Ri-2 |  |  | F: AGTGATCGGTGGTACCAGGCCTGAACAAATCAGGTCAAGAAAG  R: CTGACGTAGGGGCGATAGAGCTCTGTTTGCATTGGCTTCT |
| Os1-1 | p*OsMADS1*::*GUS* | pCAMBIA1305.1 | F: CTCGGTACCCGGGGATCCCACGACGTTACTTGAGAACCTATTC  R: CCCTCAGATCTACCATGGCTTCTTCCTCCTCCTCTCCTCTCT |

Sequences marked in red or blue colors represent add-on sequences homologous to either side of the restriction site used to linearization of the vector; sequences marked in green or purple colors represent the restriction site.
